# Supplementary material for: Identification by High-Throughput Real-Time PCR of 30 Major Circulating Listeria monocytogenes Clonal Complexes in Europe
Source: Microbiol Spectr. 2023 May 9;11(3):e03954-22. doi: 10.1128/spectrum.03954-22 (PMC10269651; doi:10.1128/spectrum.03954-22)
Supplement: Supplemental file 1 — Legends and references for Tables S1 and S2. Download spectrum.03954-22-s0001.pdf, PDF file, 0.5 MB [file spectrum.03954-22-s0001.pdf]

Supplemental legends

Table S1: Exclusivity and inclusivity strain panels used for analytical specificity and sensitivity (strain panels C and E)

Table S2: List of the 954 *Lm* (genomic panel A) strain genomes used for designing primers and probes

Supplemental references

1. Felix B, Feurer C, Maillet A, Guillier L, Boscher E, Kerouanton A, Denis M, Roussel S. 2018. Population Genetic Structure of *Listeria monocytogenes* Strains Isolated From the Pig and Pork Production Chain in France. Front Microbiol 9:684.
2. Painset A, Bjorkman JT, Kiil K, Guillier L, Mariet JF, Felix B, Amar C, Rotariu O, Roussel S, Perez-Reche F, Brisse S, Moura A, Lecuit M, Forbes K, Strachan N, Grant K, Moller-Nielsen E, Dallman TJ. 2019. LiSEQ - whole-genome sequencing of a cross-sectional survey of *Listeria monocytogenes* in ready-to-eat foods and human clinical cases in Europe. Microb Genom 5.
3. ECDC. 2019. European Centre for Disease Prevention and Control. Sixth external quality assessment scheme for *Listeria monocytogenes* typing. Stockholm: ECDC, 2019.
4. EFSA-ECDC. 2019. JOINT ECDC–EFSA RAPID OUTBREAK ASSESSMENT. Multi-country outbreak of *Listeria monocytogenes* clonal complex 8 infections linked to consumption of cold-smoked fish products.
5. Brauge T, Midelet-Bourdin G, Soumet C. 2019. Viability Detection of Foodborne Bacterial Pathogens in Food Environment by PMA-qPCR and by Microscopic Observation. Methods Mol Biol 1918:117-128.
6. Brauge T, Trigueros S, Briet A, Debuiche S, Leleu G, Gassilloud B, Wilhelm A, Py J, Midelet G. 2021. MALDI-TOF Mass Spectrometry Fingerprinting Performance Versus 16S rDNA Sequencing to Identify Bacterial Microflora From Seafood Products and Sea Water Samples. Frontiers in Marine Science 8.
7. Weller D, Andrus A, Wiedmann M, den Bakker HC. 2015. *Listeria booriae sp. nov. and Listeria newyorkensis sp. nov.,* from food processing environments in the USA. Int J Syst Evol Microbiol 65:286-292.
8. den Bakker HC, Warchocki S, Wright EM, Allred AF, Ahlstrom C, Manuel CS, Stasiewicz MJ, Burrell A, Roof S, Strawn L, Fortes ED, Nightingale KK, Kephart D, Wiedmann M. 2014. Five new species of *Listeria* (*L. floridensis sp. nov, L. aquatica sp. nov., L. cornellensis sp. nov. L. riparia sp. nov., and L. grandensis sp. nov.*) from agricultural and natural environments in the United States. Int J Syst Evol Microbiol.
9. Lang Halter E, Neuhaus K, Scherer S. 2013. *Listeria weihenstephanensis sp. nov.*, isolated from the water plant Lemna trisulca taken from a freshwater pond. Int J Syst Evol Microbiol 63:641-647.
10. Leclercq A, Clermont D, Bizet C, Grimont PAD, Le Fleche-Mateos A, Roche SM, Buchrieser C, Cadet-Daniel V, Le Monnier A, Lecuit M, Allerberger F. 2010. *Listeria rocourtiae sp. nov.* Int J Syst Evol Microbiol 60:2210-2214.
11. Bertsch D, Rau J, Eugster MR, Haug MC, Lawson PA, Lacroix C, Meile L. 2013. *Listeria fleischmannii sp. nov.,* isolated from cheese. Int J Syst Evol Microbiol 63:526-532.

12. Graves LM, Helsel LO, Steigerwalt AG, Morey RE, Daneshvar MI, Roof SE, Orsi RH, Fortes ED, Milillo SR, den Bakker HC, Wiedmann M, Swaminathan B, Sauders BD. 2010. *Listeria marthii sp. nov.,* isolated from the natural environment, Finger Lakes National Forest. Int J Syst Evol Microbiol 60:1280-1288.
