# Supplementary material for: Identification by High-Throughput Real-Time PCR of 30 Major Circulating Listeria monocytogenes Clonal Complexes in Europe
Source: Microbiol Spectr. 2023 May 9;11(3):e03954-22. doi: 10.1128/spectrum.03954-22 (PMC10269651; doi:10.1128/spectrum.03954-22)
Supplement: Supplemental file 2 — Tables S1 and S2. Download spectrum.03954-22-s0002.pdf, PDF file, 0.7 MB [file spectrum.03954-22-s0002.pdf]

Table S1 : Exclusivity and inclusivity strain panels (SP-C and E) used for analytical specificity and sensitivity

MLST clonal complexe	MLST sequence type	Subdivision markers	Panel	Collection ID	Other ID	Accession number	Origine	Country of isolation	Year of isolation	Extraction validation	High throughput	Conventional Multiplex	Fast Mutliplex	Reference
CC1	1	not performed	SP-C	02EB369LM			Milk and milk products	FR	2002		Yes			
CC1	328	CC1-SD_1	SP-C	14SEL873LM		ERR2734417	Meat and meat products	FR	Unknown		Yes			(1)
CC1	1	CC1-SD_1	SP-C & SP-E	17SEL1LM			Milk and milk products	FR	2017		Yes	Yes	Yes	
CC1	1	CC1-SD_1	SP-C	17SEL7LM		ERR4284493	Meat and meat products	FR	Unknown		Yes			(1)
CC1	1	CC1-SD_1	SP-C & SP-E	17SEL57LM			Milk and milk products	FR	2017		Yes	Yes	Yes	
CC1	308	CC1-SD_2	SP-C & SP-E	18SEL2LM			Elaborated food products combining several food categories	FR	2017		Yes	Yes	Yes	
CC1	248	CC1-SD_2	SP-C	18SEL202LM	VI 58135		Animal sample	NO	2012		Yes			
CC1	1	CC1-SD_1	SP-C	18SEL563LM		ERR4284357	Milk and milk products	FR	2018		Yes			(1)
CC1	1 (Abcz, cat, ldh missing)	CC1-SD_2	SP-C	18SEL578LM	LV1161		Animal sample	CZ	Unknown		Yes			
CC1	515	CC1-SD_2	SP-C	19SEL296LM	L1333	ERR4284593	Animal sample	HR	2019		Yes			(1)
CC1	73	CC1-SD_1	SP-C	19SEL325LM	L219	ERR4285089	Animal sample	SI	2019		Yes			(1)
CC1	1	CC1-SD_1	SP-C	19SEL422LM			Milk and milk products	FR	2019		Yes			
CC1	1	CC1-SD_1	SP-C	19SEL664LM			Milk and milk products	FR	2019		Yes			
CC1	1	CC1-SD_2	SP-C	19SEL714LM		ERR8025991	Animal sample	BE	2017		Yes			
CC1	1	CC1-SD_2	SP-C	19SEL757LM			Animal sample	BE	2017		Yes			
CC1	1	CC1-SD_2	SP-C	19SEL766LM		ERR8025974	Animal sample	BE	2017		Yes			
CC1	1	CC1-SD_2	SP-C	19SEL858LM		ERR4284557	Fruit, vegetables, cereals and herbs	FR	2019		Yes			(1)
CC1	1	CC1-SD_2	SP-C	20SEL39LM			Milk and milk products	FR	2019		Yes			
CC1	1	CC1-SD_1	SP-C	20SEL106LM			Milk and milk products	FR	2020		Yes			
CC1	1	CC1-SD_1	SP-C	20SEL138LM			Milk and milk products	FR	Unknown		Yes			
CC1	1		SP-E		2021.TE.312420.1.1		Animal sample	IT	2012				Yes	
CC1	1		SP-E		2021.TE.313140.1.1		Meat and meat products	IT	2021				Yes	
CC1	1		SP-E		2020LABTEAZ2057.A		Animal sample	IT	2020				Yes	
CC1	595		SP-E		L00326		Fish and fishery products	NL	2015				Yes	
CC1	1		SP-E		L00458		Meat and meat products	NL	2016				Yes	
CC1	1		SP-E		L00489		Meat and meat products	NL	2016				Yes	
CC1	1		SP-E		L00738		Meat and meat products	NL	2017				Yes	
CC1	1		SP-E		L01118		Meat and meat products	NL	2018				Yes	
CC1	1		SP-E		L01354		Fish and fishery products	NL	2019				Yes	
CC1	1		SP-E		L01417		Fish and fishery products	NL	2019				Yes	
CC1	145		SP-E		L01504		Ring trial material	NL	2020				Yes	
CC1	1		SP-E		L01576		Unknown	NL	2020				Yes	
CC1	1		SP-E		L01668		Meat and meat products	NL	2020				Yes	
CC1	1		SP-E		L02012		Meat and meat products	NL	2020				Yes	
CC183	183	CC1-SD_2	SP-C	15SEL805LM		SRR7441189	Milk and milk products	FR	2015		Yes			(2)
CC183	382	CC1-SD_2	SP-C	17SEL488LM			Human clinical cases ECDC EQA-5 REF 10	DK	2017		Yes			(3)
ST773	773	CC1-SD_2	SP-C	19SEL701LM			Animal sample	BE	2017		Yes			
ST213	213	CC1-SD_2	SP-C	17SEL483LM			Human clinical cases ECDC EQA-5 REF 7	DK	2017		Yes			(3)
CC2	2		SP-C	08CEB85LM		ERR4284455	Fish and fishery products	FR	2008		Yes			(1)
CC2	2		SP-C & SP-E	12CEB1097LM		ERS1374990	Fruit, vegetables, cereals and herbs	FR	2012		Yes	Yes	Yes	(1)
CC2	2		SP-C & SP-E	17SEL376LM		ERR4284468	Meat and meat products	FR	2017	Yes	Yes	Yes	Yes	(1)
CC2	2		SP-C	17SEL462LM			Meat and meat products	FR	2017		Yes			
CC2	2		SP-C & SP-E	17SEL514LM		ERR4284329	Elaborated food products combining several food categories	FR	2017		Yes	Yes	Yes	(1)
CC2	2		SP-C	18SEL547LM	H10354009201		Fruit, vegetables, cereals and herbs	UK	2018		Yes			
CC2	2		SP-C	19SEL39LM			Animal sample	SK	2018		Yes			
CC2	2		SP-C	19SEL40LM			Animal sample	SK	2018		Yes			
CC2	2		SP-C	19SEL48LM	10694/Z/2018		Fruit, vegetables, cereals and herbs	CY	2018		Yes			
CC2	145		SP-C	19SEL199LM	SLCC4139		Animal sample	AT	2019		Yes			
CC2	2		SP-C	19SEL483LM		ERR4284432	Fruit, vegetables, cereals and herbs	FR	2019		Yes			(1)
CC2	2		SP-C	19SEL488LM		ERR4284485	Fish and fishery products	FR	2019		Yes			(1)
CC2	2		SP-C	19SEL489LM		ERR4284411	Elaborated food products combining several food categories	FR	2019		Yes			(1)
CC2	2		SP-C	19SEL659LM			Environment sample	FR	2019		Yes			
CC2	2		SP-C	19SEL39LM			Animal sample	SK	2018		Yes			
CC2	2		SP-C	20SEL132LM			Milk and milk products	FR	2020		Yes			
CC2	2		SP-C	BO34		ERS1374956	Meat and meat products	FR	2000		Yes			
CC2	2		SP-E		2020.TE.215646.1.1		Meat and meat products	IT	2020				Yes	
CC2	2		SP-E		2021.TE.309493.1.2		Meat and meat products	IT	2021				Yes	
CC2	2		SP-E		2021.TE.309498.1.2		Meat and meat products	IT	2021				Yes	
CC2	2		SP-E		L00462		Meat and meat products	NL	2016				Yes	
CC2	2		SP-E		L00482		Meat and meat products	NL	2016				Yes	
CC2	2		SP-E		L00538		Meat and meat products	NL	2016				Yes	
CC2	2		SP-E		L01050		Meat and meat products	NL	2018				Yes	
CC2	145		SP-E		L01063		Ring trial material	NL	2018				Yes	
CC2	2		SP-E		L01240		Meat and meat products	NL	2019				Yes	
CC2	2		SP-E		L01558		Meat and meat products	NL	2020				Yes	
CC2	2		SP-E		L01817		Fish and fishery products	NL	2020				Yes	
CC2	2		SP-E		L01872		Meat and meat products	NL	2020				Yes	
CC3	3		SP-C	03EB237LM		ERS1375013	Meat and meat products	FR	2003		Yes			
CC3	3		SP-C	03EB411LM		ERS1375115	Elaborated food products combining several food categories	FR	2006		Yes			
CC3	3		SP-C	06CEB216LM		ERS1374965	Elaborated food products combining several food categories	FR	2006		Yes			
CC3	3		SP-C & SP-E	10CEB88LM		ERS1375030	Meat and meat products	FR	2010		Yes	Yes	Yes	
CC3	3		SP-C	18SEL66LM		ERR4284509	Elaborated food products combining several food categories	FR	2018		Yes			(1)
CC3	3		SP-C & SP-E	19SEL477LM		ERR4284487	Meat and meat products	FR	2018		Yes	Yes	Yes	(1)
CC3	3		SP-C & SP-E	19SEL490LM		ERR4284425	Elaborated food products combining several food categories	FR	2019	Yes	Yes	Yes	Yes	(1)
CC3	860		SP-C	20SEL12LM			Meat and meat products	FR	2019		Yes			
CC3	3		SP-C	20SEL21LM		ERR4284422	Elaborated food products combining several food categories	FR	2019		Yes			(1)
CC3	3		SP-C	20SEL33LM		ERR4284449	Fish and fishery products	FR	2019		Yes			(1)
CC3	3		SP-C	Lm177			Meat and meat products	FR	2005		Yes		Yes	
CC3	3		SP-E		2021.TE.293720.1.1			IT	2019				Yes	
CC3	3		SP-E		2021.TE.309503.1.2			IT	2021				Yes	
CC3	3		SP-E		2021.TE.310647.1.2			IT	2021				Yes	
CC3	3		SP-E		L01010		Fish and fishery products	NL	2018				Yes	
CC3	3		SP-E		L01049		Meat and meat products	NL	2018				Yes	
CC3	3		SP-E		L01236		Fish and fishery products	NL	2019				Yes	
CC3	3		SP-E		L01426		Meat and meat products	NL	2019				Yes	
CC3	3		SP-E		L01523		Meat and meat products	NL	2020				Yes	
CC3	3		SP-E		L01867		Meat and meat products	NL	2020				Yes	
CC3	3		SP-E		L01937		Meat and meat products	NL	2020				Yes	
CC3	3		SP-E		L01964		Meat and meat products	NL	2020				Yes	
CC3	3		SP-E		L01985		Meat and meat products	NL	2020				Yes	
CC3	3		SP-E		L02027		Meat and meat products	NL	2021				Yes	
CC1000	1000		SP-C	19SEL686LM			Animal sample	BE	2017		Yes			
CC4	4		SP-C	15SEL1257LM			Milk and milk products	FR	2015		Yes			
CC4	4		SP-C	17SEL394LM		ERR4284408	Environment sample	FR	Unknown		Yes			
CC4	4		SP-C	17SEL419LM		ERR4284379	Meat and meat products	FR	2017		Yes			(1)
CC4	4		SP-C & SP-E	17SEL428LM		ERR4284371	Milk and milk products	FR	2017		Yes	Yes	Yes	(1)
CC4	4		SP-C & SP-E	17SEL449LM			Meat and meat products	FR	2017	Yes	Yes	Yes	Yes	
CC4	4		SP-C & SP-E	17SEL543LM		ERR4284534	Environment sample	FR	2017		Yes	Yes	Yes	(1)
CC4	219		SP-C	18SEL209LM	VI 58143	ERR4284883	Animal sample	NO	2012		Yes			(1)
CC4	4		SP-C	19SEL253LM			Milk and milk products	FR	2019		Yes			
CC4	4		SP-C	19SEL259LM			Milk and milk products	FR	2019		Yes			
CC4	4		SP-C	19SEL262LM			Milk and milk products	FR	2019		Yes			
CC4	4		SP-C	19SEL405LM			Milk and milk products	FR	2019		Yes			
CC4	4		SP-C	19SEL706LM			Animal sample	BE	2017		Yes			
CC4	4		SP-C	20SEL1LM		ERR4284568	Fruit, vegetables, cereals and herbs	FR	2019		Yes			(1)
CC4	4		SP-C	20SEL64LM			Milk and milk products	FR	2020		Yes			
CC4	4		SP-E		L00610		Unknown	NL	2016				Yes	
CC4	4		SP-E		L00907		Meat and meat products	NL	2017				Yes	
CC4	4		SP-E		L00962		Ring trial material	NL	2018				Yes	
CC4	4		SP-E		L00963		Ring trial material	NL	2018				Yes	
CC4	4		SP-E		L00965		Ring trial material	NL	2018				Yes	
CC4	4		SP-E		L00969		Ring trial material	NL	2018				Yes	
CC4	4		SP-E		L00971		Ring trial material	NL	2018				Yes	
CC4	4		SP-E		L00976		Meat and meat products	NL	2018				Yes	
CC4	4		SP-E		L01224		Meat and meat products	NL	2019				Yes	
CC4	816		SP-E		L01300		Food processing environment	NL	2019				Yes	
CC4	631		SP-C	21SEL52LM	Bfr-LI-03479		Meat and meat products	DE	2021		Yes			
CC5	5		SP-C	08CEB178LM		ERS1375001	Meat and meat products	FR	2008		Yes			
CC5	5		SP-C	10CEB344LM		ERS1375103	Fruit, vegetables, cereals and herbs	FR	2010		Yes			
CC5	5		SP-C	10CEB341LM		ERS1375000	Elaborated food products combining several food categories	FR	2010		Yes			
CC5	5		SP-C	18SEL724LM		ERR4284461	Meat and meat products	FR	2018		Yes			(1)
CC5	5		SP-C & SP-E	16SEL667LM			Animal sample	FR	2012		Yes	Yes	Yes	
CC5	5		SP-C	17SEL8LM			Milk and milk products	FR	2016		Yes			
CC5	5		SP-C & SP-E	17SEL456LM		ERR4284333	Elaborated food products combining several food categories	FR	2017	Yes	Yes	Yes	Yes	(1)
CC5	5		SP-C	19SEL709LM			Animal sample	BE	Unknown		Yes			
CC5	5		SP-C	19SEL731LM			Animal sample	BE	2017		Yes			
CC5	5		SP-C	19SEL485LM		ERR4284575	Milk and milk products	FR	2019		Yes			(1)
CC5	5		SP-C & SP-E	19SEL486LM			Elaborated food products combining several food categories	FR	2019		Yes	Yes	Yes	
CC5	5		SP-C	19SEL860LM		ERR4284409	Fish and fishery products	FR	2019		Yes			(1)
CC5	5		SP-C	20SEL8LM			Meat and meat products	FR	2019		Yes			
CC5	5		SP-C	21SEL320LM			Fruit, vegetables, cereals and herbs	FR	2021		Yes		Yes	
CC5	5		SP-E		2020.TE.4381.1.1		Human clinical case	IT	2020				Yes	
CC5	5		SP-E		2021.TE.307840.1.2		Meat and meat products	IT	2021				Yes	
CC5	5		SP-E		2020.TE.89536.1.1		Human clinical case	IT	2020				Yes	
CC5	5		SP-E		L00751		Meat and meat products	NL	2017				Yes	
CC5	5		SP-E		L00958		Meat and meat products	NL	2018				Yes	
CC5	5		SP-E		L01163		Meat and meat products	NL	2019				Yes	
CC5	5		SP-E		L01198		Meat and meat products	NL	2019				Yes	
CC5	5		SP-E		L01498		Meat and meat products	NL	2020				Yes	
CC5	5		SP-E		L01636		Meat and meat products	NL	2020				Yes	
CC5	5		SP-E		L01739		Elaborated food products combining several food categories	NL	2020				Yes	
CC5	5		SP-E		L01767		Meat and meat products	NL	2020				Yes	
CC5	5		SP-E		L01833		Meat and meat products	NL	2020				Yes	
CC5	5		SP-E		L01841		Ring trial material	NL	2020				Yes	
CC6	6		SP-C	06CEB188LM		ERS1374984	Fish and fishery products	FR	1998		Yes	Yes	Yes	(1)
CC6	6		SP-C	09CEB30LM		ERR4284562	Fruit, vegetables, cereals and herbs	FR	2009		Yes			(1)
CC6	6		SP-C & SP-E	17SEL82LM			Milk and milk products	FR	2017		Yes	Yes	Yes	
CC6	6		SP-C	17SEL460LM			Milk and milk products	FR	2017		Yes			
CC6	6		SP-C & SP-E	17SEL497LM		ERR4284331	Elaborated food products combining several food categories	FR	2017	Yes	Yes			(1)
CC6	6		SP-C	18SEL91LM	4412		Fruit, vegetables, cereals and herbs	HU	2018		Yes	Yes	Yes	
CC6	6		SP-C	18SEL90LM	4408		Fruit, vegetables, cereals and herbs	HU	Unknown		Yes			
CC6	6		SP-C	19SEL882LM			Milk and milk products	FR	2019		Yes			
CC6	6		SP-C	20SEL29LM		ERR4284567	Fruit, vegetables, cereals and herbs	FR	2019		Yes			(1)
CC6	6		SP-C	20SEL49LM			Milk and milk products	FR	2019		Yes			
CC6	6		SP-C	20SEL84LM			Milk and milk products	FR	2020		Yes			
CC6	6		SP-C	20SEL89LM			Environment sample	FR	2020		Yes			
CC6	6		SP-C	20SEL382LM			Environment sample	FR	2020		Yes			
CC6	6		SP-C	21SEL58LM			Elaborated food products combining several food categories	FR	2021		Yes		Yes	
CC6	6		SP-C	Lm286			Meat and meat products	FR	2010		Yes		Yes	
CC6	6		SP-E		2021.TE.293715.1.1		Fruit, vegetables, cereals and herbs	IT	2019				Yes	
CC6	6		SP-E		2021.CB.4916.1.1		Fruit, vegetables, cereals and herbs	IT	2021				Yes	
CC6	6		SP-E		2020.TE.350388.1.1		Animal sample	IT	2020				Yes	
CC6	6		SP-E		L00807		Meat and meat products	NL	2017				Yes	
CC6	6		SP-E		L00896		Meat and meat products	NL	2017				Yes	
CC6	6		SP-E		L00916		Meat and meat products	NL	2018				Yes	
CC6	616		SP-E		L01026		Meat and meat products	NL	2018				Yes	
CC6	6		SP-E		L01396		Meat and meat products	NL	2019				Yes	
CC6	6		SP-E		L01518		Fish and fishery products	NL	2020				Yes	
CC6	6		SP-E		L01584		Meat and meat products	NL	2020				Yes	
CC6	6		SP-E		L01585		Meat and meat products	NL	2020				Yes	
CC6	6		SP-E		L01667		Food processing environment	NL	2020				Yes	
CC7	7		SP-C	09CEB444LM			Meat and meat products	FR	Unknown		Yes			
CC7	7		SP-C	11CEB447LM			Fruit, vegetables, cereals and herbs	FR	2011		Yes			
CC7	7		SP-C	15SEL41LM	2011.4267	SRR7440759	Milk and milk products	SK	Unknown		Yes			(2)
CC7	691		SP-C & SP-E	16SEL17LM			Meat and meat products	FR	2011		Yes	Yes	Yes	
CC7	7		SP-C	17SEL15LM		ERR4284428	Fish and fishery products	FR	2007		Yes			(1)
CC7	7		SP-C	17SEL102LM		ERR4284538	Environment sample	FR	2017		Yes			(1)
CC7	7		SP-C	17SEL401LM		ERR4284378	Milk and milk products	FR	2017	Yes	Yes			(1)
CC7	7		SP-C	17SEL420LM		ERR4284380	Milk and milk products	FR	2017		Yes			(1)
CC7	7		SP-C	17SEL567LM		ERR4284375	Milk and milk products	FR	2017		Yes			(1)
CC7	7		SP-C	18SEL365LM	L399		Fruit, vegetables, cereals and herbs	SI	2018		Yes			
CC7	511		SP-C & SP-E	19SEL29LM	314	ERR4285120	Animal sample	SK	2018		Yes	Yes	Yes	
CC7	107		SP-C & SP-E	19SEL164LM			Meat and meat products	FR	2019		Yes	Yes	Yes	
CC7	23		SP-C	19SEL168LM			Animal sample	FR	2019		Yes			
CC7	111		SP-C	19SEL174LM	SLCC721		Animal sample	AT	2019		Yes			
CC7	7		SP-C	19SEL177LM	SLCC878		Animal sample	AT	2019		Yes			
CC7	7		SP-C	19SEL183LM	SLCC2177		Animal sample	AT	2019		Yes			
CC7	7		SP-C	19SEL191LM	SLCC3165		Animal sample	AT	2019		Yes			
CC7	7		SP-C	19SEL194LM	SLCC3275		Animal sample	AT	2019		Yes			
CC7	461		SP-C	19SEL195LM	SLCC351		Animal sample	AT	2019		Yes			
CC7	7		SP-C	19SEL201LM	SLCC4142		Animal sample	AT	2019		Yes			
CC7	7		SP-C	20SEL9LM		ERR4284565	Fruit, vegetables, cereals and herbs	FR	2019		Yes			(1)
CC7	691		SP-C	20SEL32LM		ERR4284584	Fruit, vegetables, cereals and herbs	FR	2019		Yes			(1)
CC7	7		SP-C	20SEL103LM			Milk and milk products	FR	2020		Yes			
CC7	7		SP-C	20SEL323LM			Fruit, vegetables, cereals and herbs	FR	2020		Yes			
CC7	7		SP-C	21SEL138LM			Milk and milk products	FR	2021		Yes			
CC7	7		SP-C	Lm160			Meat and meat products	FR	2004		Yes			
CC7	7		SP-E		2021.TE.293725.1.1		Milk and milk products	IT	2019				Yes	
CC7	7		SP-E		2020.TE.349901.1.1		Fish and fishery products	IT	2020				Yes	

CC7	7		SP-E		2021.CB.4745.1.1		Animal sample	IT	2020				Yes	
CC7	7		SP-E		L01887		Meat and meat products	NL	2020				Yes	
CC7	7		SP-E		L00414		Fish and fishery products	NL	2016				Yes	
CC7	7		SP-E		L00815		Fish and fishery products	NL	2017				Yes	
CC7	7		SP-E		L00927		Fish and fishery products	NL	2018				Yes	
CC7	7		SP-E		L01145		Meat and meat products	NL	2019				Yes	
CC7	7		SP-E		L01260		Fish and fishery products	NL	2019				Yes	
CC7	7		SP-E		L01404		Fish and fishery products	NL	2019				Yes	
CC7	7		SP-E		L01462		Meat and meat products	NL	2019				Yes	
CC7	7		SP-E		L01608		Meat and meat products	NL	2020				Yes	
CC7	7		SP-E		L01911		Meat and meat products	NL	2020				Yes	
CC8	8		SP-C	14SEL15LM			Meat and meat products	FR	2014		Yes			
CC8	8		SP-C	14SEL33LM			Meat and meat products	FR	Unknown		Yes			
CC8	8		SP-C	14SEL409LM			Elaborated food products combining several food categories	FR	Unknown		Yes			
CC8	16		SP-C	15SEL707LM			Meat and meat products	FR	2015		Yes			
CC8	16		SP-C	15SEL964LM			Unspecified	FR	Unknown		Yes			
CC8	8		SP-C	16SEL517LM			Animal sample	SI	Unknown		Yes			
CC8	8		SP-C & SP-E	17SEL12LM		ERR4284498	Meat and meat products	FR	2017	Yes	Yes	Yes	Yes	(1)
CC8	16		SP-C	17SEL59LM		ERR4284327	Environment sample	FR	2017		Yes			(1)
CC8	8		SP-C & SP-E	17SEL361LM		ERR4284497	Animal sample	FR	2017		Yes	Yes	Yes	(1)
CC8	8		SP-C & SP-E	17SEL371LM		ERR4284334	Elaborated food products combining several food categories	FR	2017		Yes	Yes	Yes	(1)
CC8	8		SP-C	17SEL411LM		ERR4284417	Fish and fishery products	FR	2017		Yes			(1)
CC8	8		SP-C	18SEL63LM		ERR4284508	Meat and meat products	FR	2018		Yes			(1)
CC8	588		SP-C	18SEL216LM	VI 58152		Animal sample	NO	2012		Yes			
CC8	1247		SP-C	18SEL260LM		ERR4284440	Fish and fishery products	FR	2018		Yes			(1)
CC8	8		SP-C	18SEL306LM			Animal sample	CZ	Unknown		Yes			
CC8	551		SP-C	19SEL5LM	VI 50926		Fish and fishery products	NO	2019		Yes			
CC8	8		SP-C	19SEL49LM	10696/Z/2018		Fruit, vegetables, cereals and herbs	CY	2018		Yes			
CC8	1247		SP-C	19SEL271LM	1902057/14-5		Fish and fishery products	EE	2019		Yes			(4)
CC8	8		SP-C	19SEL479LM		ERR4284435	Fish and fishery products	FR	2019		Yes			(1)
CC8	8		SP-C	19SEL482LM		ERR4284433	Fish and fishery products	FR	2019		Yes			(1)
CC8	8		SP-C	19SEL487LM		ERR4284486	Meat and meat products	FR	2019		Yes			(1)
CC8	8		SP-C	19SEL777LM			Animal sample	BE	2017		Yes			
CC8	16		SP-C	19SEL807LM			Meat and meat products	BE	Unknown		Yes			
CC8	8		SP-C	20SEL16LM			Meat and meat products	FR	2019		Yes			
CC8	16		SP-C	20SEL201LM			Unspecified	FR	2020		Yes			
CC8	8		SP-C	21SEL325LM			Meat and meat products	FR	2021		Yes			
CC8	8		SP-C	181		ERS4774093	Fruit, vegetables, cereals and herbs	HU	2021		Yes			(1)
CC8	8		SP-C	299			Fruit, vegetables, cereals and herbs	HU	2021		Yes			
CC8	8		SP-C	379			Fruit, vegetables, cereals and herbs	HU	2021		Yes			
CC8	8		SP-C	394			Fruit, vegetables, cereals and herbs	HU	2021		Yes			
CC8	8		SP-C	Lm175			Meat and meat products	FR	2005		Yes			
CC8	8		SP-E		L01715		Meat and meat products	NL	2020				Yes	
CC8	8		SP-E		L01718		Meat and meat products	NL	2020				Yes	
CC8	8		SP-E		L01728		Meat and meat products	NL	2020				Yes	
CC8	8		SP-E		L01769		Meat and meat products	NL	2020				Yes	
CC8	8		SP-E		L01775		Meat and meat products	NL	2020				Yes	
CC8	8		SP-E		L01806		Meat and meat products	NL	2020				Yes	
CC8	8		SP-E		L01856		Fish and fishery products	NL	2020				Yes	
CC8	8		SP-E		L01859		Meat and meat products	NL	2020				Yes	
CC8	8		SP-E		L01879		Meat and meat products	NL	2020				Yes	
CC8	2117		SP-E		L01910		Meat and meat products	NL	2020				Yes	
ST1110	1110		SP-C	18SEL601LM	L347	ERR8026074	Environment sample	SI	2018		Yes			(1)
CC9	9		SP-C	14SEL987LM			Elaborated food products combining several food categories	FR	Unknown		Yes			
CC9	9		SP-C	15SEL871LM			Meat and meat products	FR	2015		Yes			
CC9	9		SP-C	15SEL1560LM	BfR-LI-01005		Meat and meat products	DE	2014		Yes			
CC9	223		SP-C	16SEL915LM			Fish and fishery products	FR	2015		Yes			
CC9	9		SP-C	16SEL1177LM	SLCC1958		Animal sample	AT	1964		Yes			
CC9	9		SP-C	16CEB1196LM	SLCC3683		Unspecified	AT	Unknown		Yes			
CC9	9		SP-C	17SEL10LM		ERR4284499	Meat and meat products	FR	2017	Yes	Yes			(1)
CC9	9		SP-C & SP-E	17SEL16LM			Fish and fishery products	FR	2017		Yes	Yes	Yes	
CC9	9		SP-C	17SEL65LM			Fish and fishery products	FR	2017		Yes			
CC9	9		SP-C & SP-E	17SEL106LM		ERR4284477	Meat and meat products	FR	2017		Yes	Yes	Yes	(1)
CC9	9		SP-C	17SEL355LM			Fish and fishery products	FR	2017		Yes			
CC9	9		SP-C	17SEL374LM		ERR4284470	Meat and meat products	FR	2017		Yes			(1)
CC9	622		SP-C & SP-E	17SEL375LM		ERR4284469	Meat and meat products	FR	2017		Yes	Yes	Yes	(1)
CC9	580		SP-C	17SEL494LM		ERR4284332	Meat and meat products	FR	2017		Yes			(1)
CC9	9		SP-C	17SEL523LM			Meat and meat products	FR	2017		Yes			
CC9	9		SP-C	17SEL509LM			Fish and fishery products	FR	2017		Yes			
CC9	9		SP-C	18SEL65LM		ERR4284510	Meat and meat products	FR	2018		Yes			(1)
CC9	9		SP-C	18SEL261LM		ERR4284492	Meat and meat products	FR	2018		Yes			(1)
CC9	9		SP-C	18SEL416LM			Animal sample	LV	Unknown		Yes			
CC9	2818		SP-C	18SEL762LM			Environment sample	FR	2018		Yes			
CC9	9		SP-C	19SEL756LM			Animal sample	BE	2017		Yes			
CC9	9		SP-C	19SEL849LM		ERR4284459	Meat and meat products	FR	2019		Yes			(1)
CC9	9		SP-C	19SEL851LM		ERR4284483	Meat and meat products	FR	2019		Yes			(1)
CC9	9		SP-C	19SEL852LM			Meat and meat products	FR	2019		Yes			
CC9	9		SP-C	19SEL877LM			Milk and milk products	FR	2019		Yes			
CC9	9		SP-C	20SEL17LM		ERR4284549	Fruit, vegetables, cereals and herbs	FR	2019		Yes			(1)
CC9	9		SP-C	20SEL228LM			Fish and fishery products	FR	2020		Yes			
CC9	9		SP-C	21SEL229LM			Meat and meat products	FR	2021		Yes			
CC9	9		SP-E		L01301		Meat and meat products	NL	2019				Yes	
CC9	9		SP-E		L01304		Meat and meat products	NL	2019				Yes	
CC9	9		SP-E		L01307		Meat and meat products	NL	2019				Yes	
CC9	9		SP-E		L01434		Meat and meat products	NL	2019				Yes	
CC9	9		SP-E		L01881		Meat and meat products	NL	2020				Yes	
CC9	580		SP-E		L01888		Meat and meat products	NL	2020				Yes	
CC9	9		SP-E		L01900		Meat and meat products	NL	2020				Yes	
CC9	9		SP-E		L01901		Meat and meat products	NL	2020				Yes	
CC9	9		SP-E		L01903		Fish and fishery products	NL	2020				Yes	
CC9	9		SP-E		L01907		Meat and meat products	NL	2020				Yes	
ST184	184		SP-C	16SEL533LM			Animal sample	SI	2016		Yes			
CC11-ST451	451		SP-C	18SEL415LM		ERR4284818	Animal sample	LV	Unknown		Yes			
CC11-ST451	451		SP-C	19SEL25LM	33	ERR4285130	Animal sample	SK	2018		Yes			(1)
CC11-ST451	451		SP-C	19SEL90LM	LV 751	ERR8026024	Environment sample	CZ	2016		Yes			(1)
CC11-ST451	451		SP-C	19SEL112LM	510	ERR4284113	Animal sample	EE	2019		Yes			(1)
CC11-ST451	451		SP-C	19SEL113LM	571-2017	ERR4284126	Animal sample	EE	2019		Yes			(1)
CC11-ST451	451		SP-C & SP-E	19SEL868LM	19SEL868LM	ERR4284573	Fruit, vegetables, cereals and herbs	FR	2019		Yes	Yes	Yes	(1)
CC11-ST451	451		SP-C & SP-E	20SEL24LM			Meat and meat products	FR	2019		Yes	Yes	Yes	
CC11-ST451	451		SP-C	20SEL277LM			Environment sample	FR	2020		Yes			
CC11-ST451	451		SP-C & SP-E	21SEL49LM	Bfr-LI-02709		Meat and meat products	DE	2021		Yes	Yes	Yes	
CC11-ST451	451		SP-C	21SEL51LM	Bfr-LI-03070		Fish and fishery products	DE	2021		Yes			
CC11-ST451	451		SP-E		L00906		Meat and meat products	NL	2017				Yes	
CC11-ST451	451		SP-E		L01061		Meat and meat products	NL	2018				Yes	
CC11-ST451	451		SP-C & SP-E		L01288		Meat and meat products	NL	2019		Yes		Yes	
CC11-ST451	451		SP-C		L01367		Meat and meat products	NL	Unknown		Yes			
CC11-ST451	451		SP-C & SP-E		L01589		Elaborated food products combining several food categories	NL	2020		Yes		Yes	
CC11-ST451	451		SP-C		L01638		Meat and meat products	NL	Unknown		Yes			
CC11-ST451	451		SP-C & SP-E		L01716		Elaborated food products combining several food categories	NL	Unknown		Yes		Yes	
CC11-ST451	451		SP-C & SP-E		L01749		Meat and meat products	NL	2020		Yes		Yes	
CC11-ST451	451		SP-E		L01800		Meat and meat products	NL	2020				Yes	
CC11-ST451	451		SP-E		L01864		Meat and meat products	NL	2020				Yes	
CC11-ST451	451		SP-E		L01875		Meat and meat products	NL	2020				Yes	
CC11-ST451	451		SP-C & SP-E		L01897		Elaborated food products combining several food categories	NL	2020		Yes		Yes	
CC11-ST451	451		SP-C & SP-E		L1524		Elaborated food products combining several food categories	NL	Unknown		Yes			
CC11-ST451	451		SP-C		L1795		Elaborated food products combining several food categories	NL	Unknown		Yes			
CC14	399		SP-C	11CEB351LM			Meat and meat products	FR	2011		Yes		Yes	
CC14	14		SP-C	12CEB1199LM		SRR7441119	Fish and fishery products	PL	2012		Yes			(2)
CC14	206		SP-C	14SEL1343LM			Milk and milk products	FR	2014		Yes			
CC14	14		SP-C	16SEL1168LM	LV640		Environment sample	CZ	2016		Yes			
CC14	206		SP-C & SP-E	17SEL349LM			Meat and meat products	FR	2017		Yes	Yes	Yes	
CC14	14		SP-C & SP-E	18SEL157LM	3513		Meat and meat products	SK	2014	Yes	Yes	Yes	Yes	
CC14	14		SP-C	18SEL156LM			Meat and meat products	FR	2015		Yes			
CC14	399		SP-C	18SEL158LM	6888		Milk and milk products	SK	2014		Yes			
CC14	14		SP-C & SP-E	18SEL159LM	8916		Unspecified	SK	2014		Yes	Yes	Yes	
CC14	206		SP-C	18SEL174LM	DAV161281		Meat and meat products	LU	2015		Yes			
CC14	399		SP-C	19SEL352LM	6 BeMa	ERR4285079	Environment sample	SI	2018		Yes			(1)
CC14	399		SP-C	19SEL306LM	L1343	ERR4284599	Animal sample	SI	2019		Yes			(1)
CC14	14		SP-C	19SEL741LM			Animal sample	BE	2017		Yes			
CC14	14		SP-C	19SEL830LM			Milk and milk products	FR	2019		Yes			
CC14	206		SP-C	20SEL142LM	2016-TE-11612-1-1	ERR4284752	Environment sample	IT	2016		Yes			(1)
CC14	14		SP-C	20SEL104LM			Milk and milk products	FR	2020		Yes			
CC14	14		SP-E		L00389		Meat and meat products	NL	2016				Yes	
CC14	14		SP-E		L00693		Meat and meat products	NL	2017				Yes	
CC14	14		SP-E		L00844		Meat and meat products	NL	2017				Yes	
CC14	14		SP-E		L00986		Meat and meat products	NL	2018				Yes	
CC14	14		SP-E		L01245		Fish and fishery products	NL	2019				Yes	
CC14	14		SP-E		L01733		Meat and meat products	NL	2020				Yes	
CC689	689	CC121-CD_2	SP-C	21SEL53LM	Bfr-LI-03506		Meat and meat products	DE	2021		Yes			
CC14	91		SP-C	12CEB1237LM		SRR7441248	Milk and milk products	PL	2012		Yes			(2)
CC14	91		SP-C	14SEL837LM			Meat and meat products	FR	2014		Yes			
CC14	91		SP-C & SP-E	15SEL30LM		SRR7441333	Milk and milk products	SK	2015		Yes	Yes	Yes	(2)
CC14	91		SP-C & SP-E	15SEL31LM		SRR7441081	Milk and milk products	SK	2015		Yes	Yes	Yes	(2)
CC14	360		SP-C	16SEL1276LM			Environment sample	USA	2002		Yes			
CC14	91		SP-C & SP-E	17SEL22LM		ERR4284537	Milk and milk products	FR	2017		Yes	Yes	Yes	(1)
CC14	160		SP-C	19SEL184LM	SLCC2267		Animal sample	AT	2019		Yes			
CC14	160		SP-C	19SEL204LM	SLCC4151		Animal sample	AT	2019		Yes			
CC14	91		SP-C	19SEL197LM	SLCC3574		Animal sample	AT	2019		Yes			
CC14	91		SP-C	19SEL212LM	SLCC4629		Animal sample	AT	2019		Yes			
CC14	91		SP-C	19SEL755LM			Animal sample	BE	2017		Yes			
CC14	91		SP-C	21SEL423LM			Fruit, vegetables, cereals and herbs	FR	2021		Yes			
CC14	91		SP-E		L00611		Meat and meat products	NL	2016				Yes	
CC14	91		SP-E		L00641		Meat and meat products	NL	2016				Yes	
CC14	91		SP-E		L00654		Meat and meat products	NL	2017				Yes	
CC14	91		SP-E		L00694		Meat and meat products	NL	2017				Yes	
CC14	91		SP-E		L00766		Other food categories	NL	2017				Yes	
CC14	91		SP-E		L00975		Meat and meat products	NL	2018				Yes	
CC14	91		SP-E		L01164		Meat and meat products	NL	2019				Yes	
CC14	91		SP-E		L01597		Meat and meat products	NL	2020				Yes	
CC18	18		SP-C	17SEL3LM		ERR4284346	Milk and milk products	FR	2017		Yes			(1)
CC18	18		SP-C & SP-E	17SEL4LM			Milk and milk products	FR	2017		Yes	Yes	Yes	
CC18	18		SP-C	17SEL465LM		ERR4284463	Meat and meat products	FR	2017		Yes			(1)
CC18	18		SP-C & SP-E	17SEL574LM			Milk and milk products	FR	2017		Yes	Yes	Yes	
CC18	18		SP-C & SP-E	17SEL575LM		ERR4284342	Milk and milk products	FR	2017	Yes	Yes	Yes	Yes	(1)
CC18	18		SP-C	18SEL138LM	976		Fruit, vegetables, cereals and herbs	FI	2018		Yes			
CC18	18		SP-C	18SEL200LM	VI 58125	ERR4284904	Animal sample	NO	2012		Yes			(1)
CC18	18		SP-C	18SEL786LM		ERR4284528	Environment sample	FR	2018		Yes			
CC18	18		SP-C	19SEL480LM		ERR4284545	Fruit, vegetables, cereals and herbs	FR	2019		Yes			(1)
CC18	18		SP-C	19SEL767LM			Animal sample	BE	2017		Yes			
CC18	18		SP-C	21SEL276LM			Milk and milk products	FR	2021		Yes			
CC18	18		SP-C	21SEL284LM			Fruit, vegetables, cereals and herbs	FR	2021		Yes			
CC18	18		SP-C	Lm292			Meat and meat products	FR	2010		Yes			
CC18	18		SP-E		L00620		Food processing environment	NL	2016				Yes	
CC18	18		SP-E		L00646		Meat and meat products	NL	2017				Yes	
CC18	18		SP-E		L00757		Meat and meat products	NL	2017				Yes	
CC18	18		SP-E		L00762		Meat and meat products	NL	2017				Yes	
CC18	18		SP-E		L00880		Meat and meat products	NL	2017				Yes	
CC18	18		SP-E		L01097		Fish and fishery products	NL	2018				Yes	
CC18	18		SP-E		L01287		Meat and meat products	NL	2019				Yes	
CC18	18		SP-E		L01669		Elaborated food products combining several food categories	NL	2020				Yes	
CC18	18		SP-E		L01773		Meat and meat products	NL	2020				Yes	
CC18	18		SP-E		L02084		Other food categories	NL	2021				Yes	
CC19-ST398-802	802		SP-C	15SEL663LM			Fish and fishery products	FR	2015		Yes			
CC19-ST398-802	398		SP-C	18SEL195LM	VI 58125	ERR4284896	Animal sample	NO	2012		Yes			(1)
CC19-ST398-802	398		SP-C	19SEL42LM	L 133-134	ERR4285133	Animal sample	SK	2018		Yes			(1)
CC19-ST398-802	398		SP-C & SP-E	20SEL264LM			Environment sample	FR	2020		Yes	Yes	Yes	
CC19-ST398-802	398		SP-C & SP-E	21SEL61LM	L1053		Meat and meat products	NL	2018		Yes	Yes	Yes	
CC19-ST398-802	398		SP-C & SP-E	21SEL232LM	164		Fruit, vegetables, cereals and herbs	HU	Unknown		Yes	Yes	Yes	
CC19-ST398-802	398		SP-C	21SEL233LM	208		Fruit, vegetables, cereals and herbs	HU	Unknown		Yes			
CC19-ST398-802	398		SP-E		L02365		Unknown	NL	2021				Yes	
CC20	20		SP-C & SP-E	08CEB286LM		ERS1375114	Milk and milk products	FR	2008		Yes	Yes	Yes	
CC20	20		SP-C	09CEB147LM		ERS1375035	Elaborated food products combining several food categories	FR	2009		Yes			
CC20	20		SP-C & SP-E	12CEB1111LM		SRR7441021	Fish and fishery products	BG	2012		Yes	Yes	Yes	(2)
CC20	20		SP-C	12CEB1112LM		SRR7440922	Fish and fishery products	BG	Unknown		Yes			(2)
CC20	20		SP-C & SP-E	12CEB1115LM		SRR7440585	Fish and fishery products	BG	2012		Yes	Yes	Yes	(2)
CC20	20		SP-C	15SEL46LM		SRR7440737	Milk and milk products	SK	Unknown		Yes			(2)
CC20	20		SP-C	17SEL73LM			Milk and milk products	FR	2016		Yes			
CC20	20		SP-C	17SEL74LM			Milk and milk products	FR	2016	Yes	Yes			
CC20	759		SP-C	18SEL194LM	VI 58123		Animal sample	NO	2012		Yes			
CC20	20		SP-C	19SEL46LM	10686/Z/2018		Fruit, vegetables, cereals and herbs	CY	2018		Yes			
CC20	20		SP-C	19SEL310LM		ERR4284609	Animal sample	SI	2019		Yes			
CC20	20		SP-C	19SEL811LM			Milk and milk products	FR	2019		Yes			
CC20	20		SP-C	20SEL102LM			Milk and milk products	FR	2020		Yes			

CC20	20		SP-C	20SEL107LM			Milk and milk products	FR	2020		Yes			
CC20	20		SP-E		L00723		Meat and meat products	NL	2017				Yes	
CC20	20		SP-E		L01033		Fish and fishery products	NL	2018				Yes	
CC20	20		SP-E		L01107		Fish and fishery products	NL	2018				Yes	
CC20	20		SP-E		L01249		Unknown	NL	2019				Yes	
CC20	20		SP-E		L01250		Unknown	NL	2019				Yes	
CC20	20		SP-E		L01625		Fish and fishery products	NL	2020				Yes	
CC20	20		SP-E		L01695		Fish and fishery products	NL	2020				Yes	
CC20	20		SP-E		L01894		Fish and fishery products	NL	2020				Yes	
CC20	20		SP-E		L02022		Fish and fishery products	NL	2020				Yes	
CC20	20		SP-E		L02063		Food processing environment	NL	2021				Yes	
CC19	19		SP-C	03EB279LM		ERS1375083	Fish and fishery products	FR	2003		Yes			
CC19	173		SP-C	12CEB1346LM			Unspecified	NL	2011		Yes			
CC19	173		SP-C	14SEL1667LM	BfR L1440		Fish and fishery products	DE	2014		Yes			
CC19	173		SP-C	14SEL1661LM	BfR L1424	SRR7440752	Fish and fishery products	DE	2014		Yes			(2)
CC21	21		SP-C	17SEL20LM		ERR4284366	Milk and milk products	FR	2017		Yes			(1)
CC21	21		SP-C & SP-E	17SEL21LM			Environment sample	FR	2017		Yes	Yes	Yes	
CC21	21		SP-C	17SEL39LM			Animal sample	SK	Unknown		Yes			
CC21	21		SP-C & SP-E	17SEL77LM			Milk and milk products	FR	2017		Yes	Yes	Yes	
CC21	21		SP-C & SP-E	17SEL88LM			Milk and milk products	FR	2017		Yes	Yes	Yes	
CC21	21		SP-C	17SEL395LM			Environment sample	FR	Unknown		Yes			
CC21	21		SP-C	18SEL602LM			Animal sample	SI	Unknown		Yes			
CC21	21		SP-C	18SEL606LM			Animal sample	SI	Unknown		Yes			
CC21	2829		SP-C	19SEL30LM			Animal sample	SK	2018		Yes			
CC21	21		SP-C	19SEL32LM	765	ERR8026077	Environment sample	SK	Unknown		Yes			(1)
CC21	21		SP-C	19SEL37LM			Animal sample	SK	2018		Yes			
CC21	21		SP-C	19SEL307LM			Animal sample	SI	2019		Yes			
CC21	21		SP-C	19SEL721LM			Animal sample	BE	Unknown		Yes			
CC21	21		SP-E		L01858		Meat and meat products	NL	2020				Yes	
CC21	Unknown		SP-E		L02364		Unknown	NL	2021				Yes	
CC403	403		SP-C	12CEB1268LM		SRR7441252	Fish and fishery products	PL	2012		Yes			(2)
CC403	403		SP-C	17SEL366LM			Milk and milk products	FR	2017		Yes			
CC403	403		SP-C	17SEL393LM			Environment sample	FR	2017		Yes			
CC403	403		SP-C	19SEL481LM		ERR4284434	Fish and fishery products	FR	2019		Yes			(1)
CC403	403		SP-C	20SEL51LM			Milk and milk products	FR	2019		Yes			
CC26	26		SP-C	04CEB500LM			Milk and milk products	FR	2004		Yes			
CC26	26		SP-C	07CEB430LM			Milk and milk products	FR	2007		Yes			
CC26	26		SP-C & SP-E	09CEB334LM		ERS1375044	Milk and milk products	FR	2009		Yes	Yes	Yes	
CC26	26		SP-C & SP-E	09CEB411LM		ERS1374959	Milk and milk products	FR	2009		Yes	Yes	Yes	(2)
CC26	26		SP-C & SP-E	15SEL817LM		SRR7440727	Meat and meat products	ES	2015		Yes	Yes	Yes	
CC26	26		SP-C	18SEL760LM		ERR8026049	Environment sample	FR	2018		Yes			
CC26	26		SP-C	19SEL425LM			Milk and milk products	FR	2019	Yes	Yes			
CC26	26		SP-C	19SEL428LM			Milk and milk products	FR	2019		Yes			
CC26	26		SP-C	19SEL511LM			Milk and milk products	FR	2019		Yes			
CC26	26		SP-C	19SEL720LM			Animal sample	BE	2017		Yes			
CC26	26		SP-C	19SEL726LM			Animal sample	BE	2017		Yes			
CC26	26		SP-C	19SEL727LM			Animal sample	BE	2017		Yes			
CC26	26		SP-C	19SEL735LM			Animal sample	BE	2017		Yes			
CC26	26		SP-C	19SEL812LM			Milk and milk products	FR	2019		Yes			
CC26	26		SP-C	19SEL832LM			Milk and milk products	FR	2019		Yes			
CC26	26		SP-C	Lm161			Meat and meat products	FR	2005		Yes			
CC26	26		SP-E		L00722		Meat and meat products	NL	2017				Yes	
CC26	26		SP-E		L01376		Fruit vegetables cereals and herbs	NL	2019				Yes	
CC26	26		SP-E		L01411		Food processing environment	NL	2019				Yes	
CC26	26		SP-E		L01655		Elaborated food products combining several food categories	NL	2020				Yes	
CC26	26		SP-E		L01661		Food processing environment	NL	2020				Yes	
CC26	26		SP-E		L01828		Fruit vegetables cereals and herbs	NL	2020				Yes	
CC26	26		SP-E		L01883		Milk and milk products	NL	2020				Yes	
CC26	26		SP-E		L01885		Elaborated food products combining several food categories	NL	2020				Yes	
CC26	26		SP-E		L01938		Elaborated food products combining several food categories	NL	2020				Yes	
CC26	26		SP-E		L01981		Elaborated food products combining several food categories	NL	2020				Yes	
CC29	849		SP-C & SP-E	17SEL50LM			Milk and milk products	FR	2017		Yes	Yes	Yes	
CC29	29		SP-C & SP-E	17SEL70LM			Milk and milk products	FR	2016	Yes	Yes	Yes	Yes	
CC29	29		SP-C	17SEL71LM			Milk and milk products	FR	2017		Yes			
CC29	29		SP-C	17SEL433LM			Milk and milk products	FR	2017		Yes			
CC29	29		SP-C & SP-E	17SEL568LM		ERR4284495	Meat and meat products	FR	2017		Yes	Yes	Yes	(1)
CC29	29		SP-C	19SEL130LM	1321	ERR4284109	Animal sample	EE	2019		Yes			(1)
CC29	29		SP-C	19SEL298LM			Animal sample	SI	2019		Yes			
CC29	849		SP-C	19SEL530LM			Milk and milk products	FR	2019		Yes			
CC29	849		SP-C	19SEL537LM			Milk and milk products	FR	2019		Yes			
CC29	29		SP-C	19SEL723LM			Animal sample	BE	2017		Yes			
CC29	29		SP-C	19SEL753LM			Animal sample	BE	2017		Yes			
CC29	849		SP-C	19SEL813LM			Milk and milk products	FR	2019		Yes			
CC29	29		SP-C	20SEL110LM			Milk and milk products	FR	2020		Yes			
CC29	29		SP-C	21SEL354LM			Milk and milk products	FR	2021		Yes			
CC29	29		SP-E		L01075		Meat and meat products	NL	2018				Yes	
CC29	29		SP-E		L01003		Meat and meat products	NL	2018				Yes	
CC31	31		SP-C & SP-E	05CEB474LM		ERS1374961	Meat and meat products	FR	2005		Yes			
CC31	31		SP-C	11CEB446LM		ERR4284558	Fruit, vegetables, cereals and herbs	FR	2011		Yes	Yes	Yes	(1)
CC31	31		SP-C	14SEL861LM			Meat and meat products	FR	2012		Yes			
CC31	325		SP-C	15SEL797LM		SRR7441124	Fish and fishery products	ES	Unknown		Yes			(2)
CC31	31		SP-C & SP-E	17SEL370LM		ERR4284506	Meat and meat products	FR	2017	Yes	Yes	Yes	Yes	(1)
CC31	31		SP-C & SP-E	19SEL381LM			Environment sample	FR	2019		Yes	Yes	Yes	
CC31	325		SP-C	19SEL850LM		ERR4284341	Milk and milk products	FR	2019		Yes			(1)
CC31	325		SP-C	18SEL548LM	H11494027701		Fruit, vegetables, cereals and herbs	UK	2018		Yes			
CC31	31		SP-C	20SEL63LM			Milk and milk products	FR	2020		Yes			
CC31	31		SP-C	20SEL13LM		ERR4284481	Milk and milk products	FR	2019		Yes			(1)
CC31	31		SP-C	20SEL61LM			Milk and milk products	FR	2020		Yes			
CC31	31		SP-C	20SEL97LM			Elaborated food products combining several food categories	FR	2020		Yes			
CC31	31		SP-C	Lm202			Unspecified	FR	Unknown		Yes			
CC31	31		SP-E		L00371		Meat and meat products	NL	2016				Yes	
CC31	31		SP-E		L00396		Fish and fishery products	NL	2016				Yes	
CC31	31		SP-E		L00796		Fish and fishery products	NL	2017				Yes	
CC31	31		SP-E		L00895		Fish and fishery products	NL	2017				Yes	
CC31	31		SP-E		L01068		Meat and meat products	NL	2018				Yes	
CC31	31		SP-E		L01192		Meat and meat products	NL	2019				Yes	
CC31	31		SP-E		L01759		Food processing environment	NL	2020				Yes	
CC31	31		SP-E		L01794		Fish and fishery products	NL	2020				Yes	
CC31	31		SP-E		L01803		Meat and meat products	NL	2020				Yes	
CC31	31		SP-E		L02023		Fish and fishery products	NL	2020				Yes	
CC37	37	CC37-CD_1	SP-C	16SEL645LM			Animal sample	FR	2012		Yes			
CC37	37	CC37-CD_1	SP-C	16SEL1269LM		ERR4284367	Milk and milk products	FR	2016		Yes			(1)
CC37	37	CC37-CD_1	SP-C	17SEL103LM		ERR4284501	Meat and meat products	FR	2017		Yes			(1)
CC37	37	CC37-CD_2	SP-C & SP-E	17SEL105LM		ERR4284500	Meat and meat products	FR	2017		Yes	Yes	Yes	(1)
CC37	37	CC37-CD_1	SP-C & SP-E	17SEL344LM		ERR4284373	Milk and milk products	FR	2017		Yes	Yes	Yes	(1)
CC37	37	CC37-CD_2	SP-C & SP-E	17SEL359LM			Milk and milk products	FR	2016		Yes	Yes	Yes	
CC37	37	CC37-CD_1	SP-C	18SEL84LM		ERR4284531	Animal sample	FR	2018		Yes			(1)
CC37	37	CC37-CD_1	SP-C	18SEL92LM		ERR4284460	Meat and meat products	FR	2018		Yes			(1)
CC37	2819	CC37-CD_2	SP-C	18SEL775LM			Environment sample	FR	Unknown		Yes			
CC37	37	CC37-CD_2	SP-C	19SEL294LM			Animal sample	SI	2019		Yes			
CC37	37	CC37-CD_2	SP-C	19SEL519LM			Milk and milk products	FR	2019		Yes			
CC37	37	CC37-CD_1	SP-C	19SEL736LM			Animal sample	BE	2017		Yes			
CC37	37	CC37-CD_1	SP-C	19SEL737LM			Animal sample	BE	2017		Yes			
CC37	37	CC37-CD_1	SP-C	19SEL752LM			Animal sample	BE	Unknown		Yes			
CC37	37	CC37-CD_2	SP-C	20SEL26LM			Meat and meat products	FR	2019		Yes			
CC37	37	CC37-CD_2	SP-C	20SEL27LM			Meat and meat products	FR	2019		Yes			
CC37	37	CC37-CD_2	SP-C	20SEL28LM		ERR4284546	Fruit, vegetables, cereals and herbs	FR	2019		Yes			(1)
CC37	37	CC37-CD_1	SP-C	20SEL42LM			Environment sample	FR	2019		Yes			
CC37	37	CC37-CD_1	SP-C	20SEL105LM			Milk and milk products	FR	2020		Yes			
CC37	1242	CC37-CD_2	SP-C	18SEL579LM	LV1162		Animal sample	CZ	Unknown		Yes			
CC37	37		SP-E		L00529		Food processing environment	NL	2016				Yes	
CC37	37		SP-E		L00823		Meat and meat products	NL	2017				Yes	
CC37	37		SP-E		L00910		Meat and meat products	NL	2018				Yes	
CC37	37		SP-E		L01182		Fish and fishery products	NL	2019				Yes	
CC37	37		SP-E		L01186		Fish and fishery products	NL	2019				Yes	
CC37	37		SP-E		L01754		Meat and meat products	NL	2020				Yes	
CC37	37		SP-E		L01863		Meat and meat products	NL	2020				Yes	
CC37	37		SP-E		L01873		Meat and meat products	NL	2020				Yes	
CC37	37		SP-E		L01878		Meat and meat products	NL	2020				Yes	
CC37	37		SP-E		L02000		Meat and meat products	NL	2020				Yes	
CC321	321	CC37-CD_2	SP-C	12CEB1206LM			Fish and fishery products	PL	2012		Yes			
CC321	321	CC37-CD_2	SP-C	16SEL1391LM			Environment sample	FR	2016		Yes			
CC321	321	CC37-CD_2	SP-C	17SEL454LM			Environment sample	FR	2017		Yes			
CC321	321	CC37-CD_2	SP-C	18SEL430LM		ERR4284439	Fish and fishery products	FR	2018		Yes			(1)
CC321	321	CC37-CD_2	SP-C	20SEL11LM		ERR4284570	Fruit, vegetables, cereals and herbs	FR	2019		Yes			(2)
CC54	54		SP-C & SP-E	19SEL881LM			Milk and milk products	FR	2019		Yes	Yes	Yes	
CC54	54		SP-C	19SEL837LM			Milk and milk products	FR	2019		Yes			
CC54	54		SP-C	19SEL865LM			Meat and meat products	FR	2019		Yes			
CC54	54		SP-C	19SEL854LM		ERR4284457	Meat and meat products	FR	2019		Yes			(1)
CC54	54		SP-C	19SEL871LM			Meat and meat products	FR	2019		Yes			
CC54	54		SP-C	20SEL260LM			Environment sample	FR	2020		Yes			
CC54	54		SP-C	20SEL259LM			Environment sample	FR	2020		Yes			
CC54	54		SP-C	20SEL292LM			Milk and milk products	FR	2020		Yes			
CC54	54		SP-C	20SEL302LM			Fruit, vegetables, cereals and herbs	FR	2020		Yes			
CC54	54		SP-C & SP-E	21SEL8LM			Environment sample	FR	2021		Yes	Yes	Yes	
CC54	54		SP-C & SP-E	21SEL14LM			Environment sample	FR	2021		Yes	Yes	Yes	
CC54	near ST54		SP-C	21SEL65LM			Environment sample	FR	2021		Yes			
CC54	54		SP-E		L01135		Meat and meat products	NL	2019				Yes	
CC59	59		SP-C & SP-E	06CEB443LM		ERS1375031	Meat and meat products	FR	2006		Yes	Yes	Yes	
CC59	59		SP-C & SP-E	07CEB456LM		ERS1374960	Fish and fishery products	FR	2007		Yes	Yes	Yes	
CC59	59		SP-C	11CEB656LM			Animal sample	FR	2011		Yes			
CC59	59		SP-C & SP-E	17SEL517LM			Milk and milk products	FR	2017	Yes	Yes	Yes	Yes	
CC59	59		SP-C	17SEL585LM		ERR4284540	Animal sample	FR	2017		Yes			(1)
CC59	2812		SP-C	19SEL679LM			Animal sample	BE	2017		Yes			
CC59	59		SP-C	19SEL772LM			Animal sample	BE	2017		Yes			
CC59	59		SP-C	19SEL866LM		ERR4284504	Meat and meat products	FR	2019		Yes			(1)
CC59	59		SP-C	21SEL227LM			Milk and milk products	FR	Unknown		Yes			
CC59	59		SP-C	21SEL228LM			Milk and milk products	FR	2021		Yes			
CC59	59		SP-C	Lm106			Animal sample	FR	2001		Yes			
CC59	59		SP-E		L00431		Meat and meat products	NL	2016				Yes	
CC59	59		SP-E		L00544		Food processing environment	NL	2016				Yes	
CC59	59		SP-E		L00778		Meat and meat products	NL	2017				Yes	
CC59	59		SP-E		L00936		Meat and meat products	NL	2018				Yes	
CC59	59		SP-E		L01020		Fish and fishery products	NL	2018				Yes	
CC59	59		SP-E		L01274		Milk and milk products	NL	2019				Yes	
CC59	59		SP-E		L01281		Milk and milk products	NL	2019				Yes	
CC59	59		SP-E		L01282		Milk and milk products	NL	2019				Yes	
CC59	59		SP-E		L01283		Milk and milk products	NL	2019				Yes	
CC77	77		SP-C	09CEB397LM		ERR4284391	Environment sample	FR	2009		Yes			(1)
CC77	77		SP-C & SP-E	09CEB593LM			Milk and milk products	FR	2009		Yes	Yes	Yes	
CC77	77		SP-C & SP-E	10CEB373LM		SRR7440861	Milk and milk products	FR	2010		Yes	Yes	Yes	
CC77	77		SP-C	15SEL65LM		SRR7440533	Meat and meat products	NL	2015		Yes			(2)
CC77	77		SP-C	15SEL1187LM			Meat and meat products	FR	2015		Yes			
CC77	77		SP-C	16SEL216LM			Meat and meat products	FR	2015		Yes			
CC77	77		SP-C	19SEL744LM			Animal sample	BE	Unknown		Yes			
CC77	77		SP-C & SP-E	20SEL40LM			Milk and milk products	FR	2019	Yes	Yes	Yes	Yes	
CC77	77		SP-C	20SEL41LM			Milk and milk products	FR	2019		Yes			
CC77	77		SP-C	21SEL258LM			Meat and meat products	FR	2020		Yes			
CC77	77		SP-E		L00507		Food processing environment	NL	2016				Yes	
CC77	77		SP-E		L00943		Meat and meat products	NL	2018				Yes	
CC77	77		SP-E		L01275		Milk and milk products	NL	2019				Yes	
CC77	77		SP-E		L01276		Milk and milk products	NL	2019				Yes	
CC77	77		SP-E		L01277		Milk and milk products	NL	2019				Yes	
CC77	77		SP-E		L01278		Milk and milk products	NL	2019				Yes	
CC77	77		SP-E		L01279		Milk and milk products	NL	2019				Yes	
CC77	77		SP-E		L01280		Milk and milk products	NL	2019				Yes	
CC77	77		SP-E		L01460		Meat and meat products	NL	2019				Yes	
CC77	77		SP-E		L01844		Ring trial material	NL	2020				Yes	
CC87	87		SP-C	12CEB1229LM		SRR7441154	Meat and meat products	PL	2012		Yes			(2)
CC87	87		SP-C & SP-E	15SEL558LM		SRR7441131	Fish and fishery products	IT	2015		Yes	Yes	Yes	
CC87	87		SP-C & SP-E	15SEL564LM		SRR7441127	Fish and fishery products	IT	2015		Yes	Yes	Yes	
CC87	87		SP-C & SP-E	17SEL107LM		ERR4284465	Meat and meat products	FR	2017		Yes	Yes	Yes	(2)
CC87	87		SP-C	17SEL391LM			Meat and meat products	FR	2017		Yes			
CC87	87		SP-C	20SEL14LM		ERR4284424	Elaborated food products combining several food categories	FR	2019	Yes	Yes			(1)
CC87	87		SP-C	21SEL282LM			Fish and fishery products	FR	2021		Yes			
CC87	87		SP-E		2021.TE.307856.1.2		Fish and fishery products	IT	2021				Yes	
CC87	87		SP-E		2020.TE.2685.1.1		Fish and fishery products	IT	2019				Yes	
CC87	87		SP-E		L00834		Meat and meat products	NL	2017				Yes	
CC87	87		SP-E		L01027		Meat and meat products	NL	2018				Yes	
CC87	87		SP-E		L01684		Meat and meat products	NL	2020				Yes	
CC87	87		SP-E		L01685		Meat and meat products	NL	2020				Yes	
CC87	87		SP-E		L01691		Meat and meat products	NL	2020				Yes	
CC87	87		SP-E		L01692		Meat and meat products	NL	2020				Yes	

CC87	87		SP-E		L01719		Meat and meat products	NL	2020				Yes	
CC87	87		SP-E		L01720		Meat and meat products	NL	2020				Yes	
CC87	87		SP-E		L01896		Meat and meat products	NL	2020				Yes	
CC87	87		SP-E		L01683		Meat and meat products	NL	2020				Yes	
CC88	296		SP-C	20SEL140LM	2015.TE.21231.1.41	ERR4284662	Fish and fishery products	IT	2011		Yes			(1)
CC101	101		SP-C & SP-E	03EB425LM			Fish and fishery products	FR	2003		Yes	Yes	Yes	
CC101	101		SP-C	10CEB585LM		SRR7440657	Milk and milk products	FR	Unknown		Yes			(2)
CC101	101		SP-C	12CEB866LM		SRR7440963	Fish and fishery products	BE	Unknown		Yes			(2)
CC101	101		SP-C	12CEB1322LM	11-123	SRR7440565	Fish and fishery products	EE	Unknown		Yes			(2)
CC101	101		SP-C	18SEL141LM	980		Fruit, vegetables, cereals and herbs	FI	2018		Yes			
CC101	101		SP-C & SP-E	18SEL144LM	984		Fruit, vegetables, cereals and herbs	FI	2018	Yes	Yes	Yes	Yes	
CC101	101		SP-C	18SEL173LM			Elaborated food products combining several food categories	FR	2018		Yes			
CC101	431		SP-C & SP-E	19SEL861LM		ERR4284574	Fruit, vegetables, cereals and herbs	FR	2019		Yes	Yes	Yes	(1)
CC101	431		SP-C	20SEL37LM		ERR4284566	Fruit, vegetables, cereals and herbs	FR	2019		Yes			(1)
CC101	101		SP-C	20SEL218LM			Fruit, vegetables, cereals and herbs	FR	Unknown		Yes			
CC101	101		SP-C	20SEL278LM			Milk and milk products	FR	2020		Yes			
CC101	101		SP-C	21SEL252LM			Fruit, vegetables, cereals and herbs	FR	Unknown		Yes			
CC101	101		SP-C	21SEL278LM			Fruit, vegetables, cereals and herbs	FR	Unknown		Yes			
CC101	101		SP-E		2020.TE.118663.1.1		Human clinical case	IT	2020				Yes	
CC101	101		SP-E		2021.TE.293717.1.1		Meat and meat products	IT	2018				Yes	
CC101	431		SP-E		L00381		Meat and meat products	NL	2016				Yes	
CC101	431		SP-E		L00480		Meat and meat products	NL	2016				Yes	
CC101	431		SP-E		L00485		Meat and meat products	NL	2016				Yes	
CC101	431		SP-E		L00710		Meat and meat products	NL	2017				Yes	
CC101	101		SP-E		L00849		Fish and fishery products	NL	2017				Yes	
CC101	431		SP-E		L01057		Meat and meat products	NL	2018				Yes	
CC101	101		SP-E		L01132		Meat and meat products	NL	2019				Yes	
CC101	101		SP-E		L01326		Elaborated food products combining several food categories	NL	2019				Yes	
CC101	101		SP-E		L01961		Elaborated food products combining several food categories	NL	2020				Yes	
CC101	101		SP-E		L02031		Meat and meat products	NL	2021				Yes	
CC121	121	CC121-SD_2	SP-C	09CEB575LM		ERS1375135	Fish and fishery products	FR	Unknown		Yes			
CC121	121	CC121-SD_1	SP-C	11CEB362LM			Milk and milk products	FR	Unknown		Yes			
CC121	121	CC121-SD_2	SP-C	12CEB1182LM		ERS1375048	Fruit, vegetables, cereals and herbs	FR	2012		Yes			(1)
CC121	121	CC121-SD_1	SP-C	15SEL1681LM			Elaborated food products combining several food categories	FR	2013		Yes			
CC121	121	CC121-SD_2	SP-C	17SEL25LM		ERR4284507	Meat and meat products	FR	2017		Yes			(1)
CC121	121	CC121-SD_1	SP-C & SP-E	17SEL63LM		ERR4284326	Elaborated food products combining several food categories	FR	2017	Yes	Yes	Yes	Yes	(1)
CC121	121	CC121-SD_2	SP-C	17SEL104LM		ERR4284466	Animal sample	FR	2017		Yes			(1)
CC121	121	CC121-SD_2	SP-C & SP-E	17SEL369LM		ERR4284541	Fish and fishery products	FR	2017		Yes	Yes	Yes	(1)
CC121	121	CC121-SD_2	SP-C & SP-E	17SEL377LM		ERR4284472	Meat and meat products	FR	2017		Yes	Yes	Yes	(1)
CC121	121	CC121-SD_2	SP-C	17SEL516LM		ERR4284462	Meat and meat products	FR	2017		Yes			(1)
CC121	121	CC121-SD_1	SP-C	17SEL521LM		ERR4284475	Meat and meat products	FR	2017		Yes			(1)
CC121	121	CC121-SD_2	SP-C	18SEL61LM		ERR4284427	Fish and fishery products	FR	2018		Yes			(1)
CC121	121	CC121-SD_2	SP-C	18SEL150LM			Fruit, vegetables, cereals and herbs	FR	Unknown		Yes			
CC121	121	CC121-SD_1	SP-C	18SEL546LM	H10298013801		Fruit, vegetables, cereals and herbs	UK	2018		Yes			
CC121	121	CC121-SD_1	SP-C	18SEL989LM		ERR4284324	Elaborated food products combining several food categories	FR	2018		Yes			(1)
CC121	236	CC121-SD_2	SP-C	19SEL853LM		ERR4284458	Meat and meat products	FR	2019		Yes			(1)
CC121	121	CC121-SD_1	SP-C	20SEL15LM		ERR4284423	Elaborated food products combining several food categories	FR	2019		Yes			(1)
CC121	121	CC121-SD_2	SP-C	20SEL22LM		ERR4284548	Fruit, vegetables, cereals and herbs	FR	2019		Yes			(1)
CC121	121	CC121-SD_1	SP-C	20SEL23LM		ERR4284421	Elaborated food products combining several food categories	FR	2019		Yes			(1)
CC121	121	CC121-SD_2	SP-C	20SEL34LM			Meat and meat products	FR	2019		Yes			
CC121	121	CC121-SD_1	SP-C	20SEL38LM		ERR4284337	Elaborated food products combining several food categories	FR	2019		Yes			(1)
CC121	121	CC121-SD_1	SP-C	20SEL93LM			Fish and fishery products	FR	2020		Yes			
CC121	121	CC121-SD_2	SP-C	20SEL96LM			Fish and fishery products	FR	2020		Yes			
CC121	121	CC121-SD_1	SP-C	20SEL196LM			Unspecified	FR	2020		Yes			
CC121	121	CC121-SD_1	SP-C	21SEL322LM			Fish and fishery products	FR	2021		Yes			
CC121	121	CC121-SD_1	SP-C	21SEL405LM			Fruit, vegetables, cereals and herbs	FR	2021		Yes			
CC121	121	CC121-SD_2	SP-C	Lm141			Unspecified	FR	Unknown		Yes			
CC121	275	CC121-SD_2	SP-C	Lm149			Meat and meat products	FR	2005		Yes			
CC121	121		SP-E		L00294		Fish and fishery products	NL	2015				Yes	
CC121	121		SP-E		L00301		Fish and fishery products	NL	2015				Yes	
CC121	121		SP-E		L00351		Elaborated food products combining several food categories	NL	2015				Yes	
CC121	121		SP-E		L00451		Meat and meat products	NL	2016				Yes	
CC121	121		SP-E		L00463		Meat and meat products	NL	2016				Yes	
CC121	121		SP-E		L00672		Meat and meat products	NL	2017				Yes	
CC121	121		SP-E		L01502		Meat and meat products	NL	2020				Yes	
CC121	121		SP-E		L01604		Meat and meat products	NL	2020				Yes	
CC121	121		SP-E		L01768		Fish and fishery products	NL	2020				Yes	
CC121	121		SP-E		L01782		Elaborated food products combining several food categories	NL	2020				Yes	
CC155	155		SP-C	03EB246LM		ERS1375025	Elaborated food products combining several food categories	FR	2006		Yes			
CC155	155		SP-C	06CEB306LM			Meat and meat products	FR	2006		Yes			
CC155	155		SP-C	08CEB181LM		ERS1374994	Fish and fishery products	FR	2008		Yes			(1)
CC155	155		SP-C	12CEB875LM			Milk and milk products	FR	Unknown		Yes			
CC155	155		SP-C	12CEB1103LM		SRR7441027	Fish and fishery products	LV	2011		Yes			(2)
CC155	155		SP-C	12CEB1291LM			Milk and milk products	FR	Unknown		Yes			
CC155	155		SP-C	14SEL34LM			Meat and meat products	FR	Unknown		Yes			
CC155	155		SP-C	14SEL810LM		ERR4284446	Fish and fishery products	FR	Unknown		Yes			(1)
CC155	155		SP-C	14SEL986LM		ERR4284418	Fish and fishery products	FR	Unknown		Yes			(1)
CC155	155		SP-C	14SEL1423LM			Fish and fishery products	FR	Unknown		Yes			
CC155	155		SP-C	16SEL1312LM			Fish and fishery products	FR	2016		Yes			
CC155	155		SP-C & SP-E	17SEL412LM		ERR4284416	Fish and fishery products	FR	2017		Yes	Yes	Yes	(1)
CC155	155		SP-C & SP-E	18SEL258LM		ERR4284502	Meat and meat products	FR	2018		Yes	Yes	Yes	(1)
CC155	2827		SP-C	18SEL769LM			Environment sample	FR	2018		Yes			
CC155	155		SP-C & SP-E	19SEL160LM			Environment sample	FR	2019		Yes	Yes	Yes	
CC155	155		SP-C	20SEL78LM			Elaborated food products combining several food categories	FR	2020		Yes			
CC155	155		SP-C	20SEL184LM			Fish and fishery products	FR	2020		Yes			
CC155	155		SP-C	20SEL185LM			Fish and fishery products	FR	2020		Yes			
CC155	155		SP-C	20SEL193LM			Fish and fishery products	FR	2020		Yes			
CC155	155		SP-C	20SEL227LM			Meat and meat products	FR	2020		Yes			
CC155	155		SP-C	Lm157			Meat and meat products	FR	2005		Yes			
CC155	155		SP-C	Lm159			Meat and meat products	FR	2005		Yes			
CC155	155		SP-E		L00375		Meat and meat products	NL	2016				Yes	
CC155	155		SP-E		L00957		Meat and meat products	NL	2018				Yes	
CC155	155		SP-E		L01079		Meat and meat products	NL	2018				Yes	
CC155	155		SP-E		L01205		Fish and fishery products	NL	2019				Yes	
CC155	155		SP-E		L01398		Food processing environment	NL	2019				Yes	
CC155	155		SP-E		L01578		Fish and fishery products	NL	2020				Yes	
CC155	155		SP-E		L01698		Meat and meat products	NL	2020				Yes	
CC155	155		SP-E		L01699		Meat and meat products	NL	2020				Yes	
CC155	155		SP-E		L01913		Food processing environment	NL	2020				Yes	
CC155	155		SP-E		L02046		Meat and meat products	NL	2021				Yes	
CC193	662		SP-C	01EB168LM		ERS1375105	Fish and fishery products	FR	2001		Yes			
CC193	193		SP-C	06CEB178LM		ERS1375045	Fish and fishery products	FR	Unknown		Yes			
CC193	193		SP-C	06CEB180LM		ERS1374976	Fish and fishery products	FR	Unknown		Yes			
CC193	193		SP-C	08CEB03LM		ERS1375073	Meat and meat products	FR	Unknown		Yes			
CC193	193		SP-C & SP-E	11CEB245LM		ERS1375091	Fish and fishery products	FR	2011		Yes	Yes	Yes	
CC193	193		SP-C	12CEB864LM	TA1024737	SRR7441139	Fish and fishery products	EE	Unknown		Yes			(2)
CC193	193		SP-C	12CEB910LM		SRR7440931	Fish and fishery products	SK	2012		Yes			(2)
CC193	193		SP-C	12CEB1208LM		SRR7441122	Fish and fishery products	PL	Unknown		Yes			(2)
CC193	193		SP-C	20SEL10LM			Meat and meat products	FR	2019		Yes			
CC193	193		SP-C & SP-E	IN1		ERS1374979	Milk and milk products	FR	2000		Yes	Yes	Yes	
CC193	193		SP-C & SP-E	IN10		ERS1374997	Milk and milk products	FR	2000		Yes	Yes	Yes	
CC193	193		SP-E		L00295		Fish and fishery products	NL	2015				Yes	
CC193	193		SP-E		L00302		Fish and fishery products	NL	2015				Yes	
CC193	193		SP-E		L00680		Fish and fishery products	NL	2017				Yes	
CC193	196		SP-E		L00788		Ring trial material	NL	2017				Yes	
CC193	196		SP-E		L00789		Ring trial material	NL	2017				Yes	
CC193	196		SP-E		L00790		Ring trial material	NL	2017				Yes	
CC193	196		SP-E		L00791		Ring trial material	NL	2017				Yes	
CC124	124		SP-C	21SEL54LM	Bfr-LI-03646		Meat and meat products	DE	2021		Yes			
CC124	124		SP-C	19SEL70LM	L00511	ERR8026069	Animal sample	NL	2019		Yes			(1)
CC199	230		SP-C & SP-E	16SEL689LM			Animal sample	FR	2013		Yes	Yes	Yes	
CC199	199		SP-C	19SEL124LM	190	ERR4284121	Animal sample	EE	2019		Yes			(1)
CC199	199		SP-C & SP-E	20SEL79LM			Milk and milk products	FR	2020		Yes	Yes	Yes	
CC199	199		SP-C	21SEL62LM	L466		Meat and meat products	NL	2016		Yes			
CC199	199		SP-C	21SEL63LM	L479		Meat and meat products	NL	2016		Yes			
CC199	199		SP-C	21SEL47LM	Bfr-LI-02585		Meat and meat products	DE	2021		Yes			
CC199	199		SP-C	21SEL50LM	Bfr-LI-02679		Environment sample	DE	2021		Yes			
CC199	2079		SP-C	Sample 12			Meat and meat products	NL	2020		Yes			
CC199	199		SP-C	L1527	L1527		Meat and meat products	NL	Unknown		Yes			
CC199	230		SP-E		L00479		Meat and meat products	NL	2016				Yes	
CC199	230		SP-E		L00673		Meat and meat products	NL	2017				Yes	
CC199	230		SP-E		L00736		Meat and meat products	NL	2017				Yes	
CC199	230		SP-E		L00832		Meat and meat products	NL	2017				Yes	
CC199	230		SP-E		L00862		Meat and meat products	NL	2017				Yes	
CC199	230		SP-E		L00884		Meat and meat products	NL	2017				Yes	
CC199	230		SP-E		L00923		Meat and meat products	NL	2018				Yes	
CC199	230		SP-E		L00940		Meat and meat products	NL	2018				Yes	
CC199	2079		SP-E		L01527		Meat and meat products	NL	2020				Yes	
CC199	230		SP-E		L01857		Meat and meat products	NL	2020				Yes	
CC204	204		SP-C & SP-E	11CEB450LM		ERS1375009	Environment sample	FR	2011		Yes	Yes	Yes	
CC204	204		SP-C	11CEB216LM		ERR4284585	Elaborated food products combining several food categories	FR	2011		Yes			(1)
CC204	204		SP-C & SP-E	12CEB3LM			Fish and fishery products	FR	Unknown		Yes	Yes	Yes	
CC204	204		SP-C	16SEL760LM	FSL S4-0880		Environment sample	USA	Unknown		Yes			
CC204	204		SP-C	17SEL373LM			Meat and meat products	FR	2017		Yes			
CC204	204		SP-C	18SEL530LM			Meat and meat products	FR	2018		Yes			
CC204	204		SP-C & SP-E	17SEL510LM			Meat and meat products	FR	2017	Yes	Yes	Yes	Yes	
CC204	204		SP-C	19SEL759LM			Animal sample	BE	2017		Yes			
CC204	204		SP-C	20SEL36LM		ERR4284551	Fruit, vegetables, cereals and herbs	FR	2019		Yes			(1)
CC204	204		SP-C	Lm156			Meat and meat products	FR	2005		Yes			
CC204	204		SP-C	Lm339			Meat and meat products	FR	2015		Yes			
CC204	204		SP-E		L00486		Meat and meat products	NL	2016				Yes	
CC204	204		SP-C	22SEL521LM	L00500		Fish and fishery products	NL	2016		Yes		Yes	
CC204	204		SP-E		L00889		Meat and meat products	NL	2017				Yes	
CC204	204		SP-C	22SEL522LM	L01157		Meat and meat products	NL	2019		Yes		Yes	
CC204	204		SP-E		L01267		Fruit vegetables cereals and herbs	NL	2019				Yes	
CC204	204		SP-E		L01439		Elaborated food products combining several food categories	NL	2019				Yes	
CC204	204		SP-E		L01600		Meat and meat products	NL	2020				Yes	
CC204	204		SP-E		L01671		Food processing environment	NL	2020				Yes	
CC204	204		SP-E		L01675		Fish and fishery products	NL	2020				Yes	
CC224	224		SP-C & SP-E	10CEB615LM		ERS1374999	Meat and meat products	FR	2010		Yes	Yes	Yes	
CC224	224		SP-C	16SEL259LM			Meat and meat products	FR	2015		Yes			
CC224	224		SP-C	16SEL539LM	L794	ERR4285116	Animal sample	SI	2016		Yes			(1)
CC224	224		SP-C	16SEL535LM	L777	ERR4285101	Animal sample	SI	2016		Yes			(1)
CC224	386		SP-C	16SEL662LM			Animal sample	FR	2012		Yes			
CC224	224		SP-C	17SEL397LM			Environment sample	FR	2017		Yes			
CC224	224		SP-C	19SEL520LM			Environment sample	FR	2019		Yes			
CC224	224		SP-C	19SEL696LM			Animal sample	BE	Unknown		Yes			
CC224	224		SP-C & SP-E	20SEL86LM			Milk and milk products	FR	2020	Yes	Yes	Yes	Yes	
CC224	224		SP-C & SP-E	Lm105			Animal sample	FR	2001		Yes	Yes	Yes	
CC224	224		SP-E		L00471		Meat and meat products	NL	2016				Yes	
CC224	224		SP-E		L00478		Meat and meat products	NL	2016				Yes	
CC224	224		SP-E		L00597		Meat and meat products	NL	2016				Yes	
CC224	224		SP-E		L00644		Unknown	NL	2016				Yes	
CC224	224		SP-E		L01140		Meat and meat products	NL	2019				Yes	
CC224	224		SP-E		L01142		Meat and meat products	NL	2019				Yes	
CC224	224		SP-E		L01495		Meat and meat products	NL	2020				Yes	
CC224	224		SP-E		L01564		Meat and meat products	NL	2020				Yes	
CC224	224		SP-E		L01694		Fish and fishery products	NL	2020				Yes	
CC224	224		SP-E		L01928		Meat and meat products	NL	2020				Yes	
ST581	581		SP-C	18SEL599LM	L343	ERR8026073	Environment sample	SI	2018		Yes			(1)
ST585	585		SP-C	21SEL57LM	Bfr-LI-07325		Meat and meat products	DE	2021		Yes			
Salmonella enterica subsp. enterica serovar Dublin			SP-C	2019LSAL04743				FR	Unknown		Yes			
Salmonella Gallen			SP-C	2019LSAL04089				FR	Unknown		Yes			
Salmonella Reading			SP-C	2020LSAL00107				FR	Unknown		Yes			
Salmonella enterica subsp. enterica serovar Infantis			SP-C	2020LSAL00701				FR	Unknown		Yes			
Salmonella enterica subsp. enterica serovar Enteritidis			SP-C	2020LSAL00190				FR	Unknown		Yes			
Staphylococcus aureus			SP-C	05CEB18STA				FR	Unknown		Yes			
Staphylococcus aureus			SP-C	339E				FR	Unknown		Yes			
Staphylococcus aureus			SP-C	352E				FR	Unknown		Yes			
Staphylococcus aureus			SP-C	09CEB329STA				FR	Unknown		Yes			
Staphylococcus aureus			SP-C	13CEB193STA				FR	Unknown		Yes			
Clostridium perfringens			SP-C	16SBCL940				FR	Unknown		Yes			
Clostridium perfringens			SP-C	16SBCL624				FR	Unknown		Yes			
Clostridium perfringens			SP-C	16SBCL1126				FR	Unknown		Yes			
Clostridium perfringens			SP-C	17SBCL335				FR	Unknown		Yes			
Clostridium perfringens			SP-C	16SBCL525				FR	Unknown		Yes			
Pantoea agglomerans			SP-C	1V2	1V2		Fish and fishery products	FR	Unknown		Yes			(5)
Citrobacter gillenii			SP-C	P3AV1	P3AV1		Fish and fishery products	FR	Unknown		Yes			(5)
Pseudomonas fluorescens			SP-C	4C29	4C29		Fish and fishery products	FR	Unknown		Yes			(5)
Carnobacterium maltaromaticum			SP-C	P1AV5	P1AV5		Fish and fishery products	FR	Unknown		Yes			(5)
Carnobacterium divergens			SP-C	P9AV5	P9AV5		Fish and fishery products	FR	Unknown		Yes			(5)
Brochothrix thermosphacta			SP-C	16LiM83	16LiM83		Fish and fishery products	FR	Unknown		Yes			(6)
Serratia proteamaculans			SP-C	16LiP98	16LiP98		Fish and fishery products	FR	Unknown		Yes			(6)

Candida zeylanoides				SP-C	17LiF141	17LiF141		Fish and fishery products	FR	Unknown		Yes			(6)
Lactococcus lactis				SP-C	17LiF95	17LiF95		Fish and fishery products	FR	Unknown		Yes			(6)
Staphylococcus warneri				SP-C	17LiM212	17LiM212		Fish and fishery products	FR	Unknown		Yes			(6)
Listeria booria				SP-C	15SEL713LM		JNFA00000000		USA	2015		Yes			(7)
Listeria newyorkensis				SP-C	15SEL714LM		JNFB00000000		USA	2015		Yes			(7)
Listeria riparia				SP-C	15SEL741LM		JX961638		USA	2015		Yes			(8)
Listeria grandensis				SP-C	15SEL742LM		JX961635		USA	2015		Yes			(8)
Listeria weihenstephanensis				SP-C	15SEL748LM		FR850019		DE	2015		Yes			(9)
Listeria weihenstephanensis				SP-C	15SEL749LM		FR850020		DE	Unknown		Yes			(9)
Listeria rocourtiae				SP-C	15SEL743LM		FJ557241		AT	2015		Yes			(10)
Listeria aquatica				SP-C	15SEL739LM		JX961637		USA	2015		Yes			(8)
Listeria floridensis				SP-C	15SEL738LM		JX961636		USA	2015		Yes			(8)
Listeria Fleischmannii subsp. Fleischmanii				SP-C	15SEL745LM		ALWW01000001		CH	2015		Yes			(11)
Listeria fleischmannii subsp. Coloradonensis				SP-C	15SEL746LM		JQ287762		USA	2015		Yes			(8)
Listeria Fleischmannii subsp. Fleischmanii				SP-C	15SEL747LM		JN093103		CH	2015		Yes			(11)
Listeria marthii				SP-C	15SEL744LM		EU545982		USA	2015		Yes			(12)
Listeria welshimeri	2074			SP-C	19SEL330LM	INRA12462		Environment sample	FR	2019		Yes			
Listeria ivanovii	883			SP-C	19SEL800LM			Animal sample	BE	2017		Yes			
Listeria ivanovii				SP-C	18SEL757LM	OZ10_E2 / INRA12352		Environment sample	FR	2018		Yes			
Listeria seeligeri	1083			SP-C	19SEL343LM	BD18 / INRA12475		Environment sample	SI	2018		Yes			
Listeria seeligeri	1583			SP-C	18SEL790LM	SUEDE F1_E1 / INRA12381		Environment sample	SE	2018		Yes			
Listeria seeligeri	1083			SP-C	18SEL791LM	VB1.2_E2 / INRA12440		Environment sample	FR	2018		Yes			
Listeria innocua				SP-C	09CEB651LM			Meat and meat products	FR	Unknown		Yes			
Listeria innocua	1085			SP-C	19SEL222LM			Milk and milk products	FR	2019		Yes			
Listeria innocua	599			SP-C	19SEL338LM	BEMA7 / INRA12469		Environment sample	SI	2018		Yes			
Listeria innocua	1480			SP-C	19SEL634LM	AUX8 / INRA12127		Environment sample	FR	2018		Yes			
Listeria innocua	1087			SP-C	19SEL732LM			Animal sample	BE	2017		Yes			
Listeria innocua	140			SP-C	19SEL355LM	BL2121 / INRA12730		Environment sample	FR	2018		Yes			
Listeria innocua	527			SP-C	19SEL347LM	BD16 / INRA12722		Environment sample	SI	2018		Yes			
Listeria innocua	43			SP-C	19SEL26LM	176		Animal sample	SK	Unknown		Yes			
CC388	388			SP-C	17SEL399LM			Milk and milk products	FR	2017		Yes			
CC388	388			SP-C	17SEL550LM			Fruit, vegetables, cereals and herbs	FR	2017		Yes			
CC388	388			SP-C	19SEL870LM		ERR4284571	Fruit, vegetables, cereals and herbs	FR	2019	Yes	Yes			(1)
CC388	388			SP-C	19SEL869LM		ERR4284572	Fruit, vegetables, cereals and herbs	FR	2019	Yes	Yes			(1)
CC200	200			SP-C	17SEL52LM		ERR4284370	Milk and milk products	FR	2017		Yes			(1)
CC200	200			SP-C	17SEL498LM		ERR4284330	Elaborated food products combining several food categories	FR	2017		Yes			(1)
CC200	200			SP-C	11CEB340LM		ERR4284581	Fruit, vegetables, cereals and herbs	FR	2011		Yes			(1)
CC200	200			SP-C	15SEL51LM		SRR7440733	Milk and milk products	FR	2015		Yes			(2)
CC412	412			SP-C	16SEL708LM			Animal sample	FR	2014		Yes			
CC412	620			SP-C	08CEB244LM		ERS1375067	Milk and milk products	FR	2008	Yes	Yes			
ST36	36			SP-C	18SEL148LM			Elaborated food products combining several food categories	FR	2018	Yes	Yes			
ST191	191			SP-C	19SEL052LM			Fruit, vegetables, cereals and herbs	FR	Unknown		Yes			
CC207	207			SP-C	19SEL810LM			Milk and milk products	FR	2019		Yes			
CC207	207			SP-C	20SEL365LM			Meat and meat products	FR	2020		Yes			
CC207	207			SP-C	21SEL76LM			Meat and meat products	FR	2021		Yes			
ST32	32			SP-C	19SEL218LM			Milk and milk products	FR	2019		Yes			
CC415	394			SP-C	19SEL627LM	ABR3 / INRA12461		Environment sample	FR	2018		Yes			
CC412	412			SP-C	19SEL875LM			Milk and milk products	FR	2019		Yes			
CC207	695			SP-C	19SEL234LM			Milk and milk products	FR	2019		Yes			
CC475	504			SP-C	18SEL434LM		ERR4284467	Meat and meat products	FR	2018		Yes			(1)
ST517	517			SP-C	12CEB881LM		ERR4284353	Milk and milk products	FR	2012		Yes			(1)
CC177	177			SP-C	14SEL1671LM	BfR L1595	SRR7440744	Meat and meat products	DE	2014		Yes			(2)
ST13	13			SP-C	IN6		ERS1375074	Milk and milk products	FR	Unknown		Yes			
ST191	191			SP-C		Lm268		Unspecified	FR	Unknown		Yes			
CC217	217			SP-C	18SEL218LM	VI 58154	ERR4284892	Animal sample	NO	2012		Yes			(1)
CC11	11			SP-C		Lm201		Unspecified	FR	Unknown		Yes			
CC11	11			SP-C	20SEL332LM			Meat and meat products	FR	2020		Yes			
CC389	389			SP-C	19SEL722LM			Animal sample	BE	2017		Yes			
CC220	220			SP-C	05CEB573LM		ERS1374993	Fish and fishery products	FR	2005		Yes			
CC70	70			SP-C	19SEL290LM	L1327	ERR4284588	Animal sample	SI	2019		Yes			(1)
ST570	570			SP-C	19SEL292LM	L1329	ERR4284589	Animal sample	SI	2019		Yes			(1)
CC195	195			SP-C	20SEL136LM	LV79		Human clinical case	CZ	2020		Yes			
ST666	666			SP-C	18SEL761LM	OUCH73_E2 / INRA12345		Environment sample	FR	2018		Yes			
CC11	11			SP-C	20SEL151LM			Milk and milk products	FR	2020		Yes			
CC288	288			SP-C	21SEL48LM	Bfr-LI-02663		Milk and milk products	DE	2021		Yes			
CC573	573			SP-C	21SEL55LM	Bfr-LI-03883		Milk and milk products	DE	2021		Yes			
CC203	near ST203			SP-C	21SEL56LM	BfR-LI-07305		Fish and fishery products	DE	2021		Yes			
CC379	379			SP-C	20SEL355LM			Meat and meat products	FR	2020		Yes			

Table S2: List of the 954 *Listeria monocytogenes* strain genomes (GP-A) used for designing the primers and probes

Clonal complex	Sequence type	Genome reference: accession, assembly, ANSES ID	Initial ID	Origin
CC1	ST1421	SRR6829253		South Africa - not provided
CC1	ST876	SRR6829229		South Africa - not provided
CC1	ST876	SRR5099195		USA - not provided - 2016-11
CC1	ST1097	SRR2585421		Peru - avocado pulp - 41116
CC1	ST1	SRR1654984		Germany - human
CC1	ST1	ERR2261490		Germany - human listeriosis
CC1	ST1	SRR1656987		Austria - sausage (made of raw meat)
CC1	ST1	SRR1656996		Austria - sheep (brain)
CC1	ST1	SRR3707731		Canada - Coleslaw
CC1	ST1	SRR5764859		Canada:British Columbia - food processing environment
CC1	ST1	SRR3395016		Chile:Santiago - Environment
CC1	ST1	SRR6475347		Chile - frozen coho ikura (roe)
CC1	ST1	SRR6930071		Denmark - smoked salmon
CC1	ST595	ERR2523698		Denmark - not provided - 2010
CC1	ST416	ERR2512046		Denmark - not provided - 2011
CC1	ST308	SRR6366512		India - Frozen grated coconut - 43045
CC1	ST308	SRR7164131		United Kingdom - human - 2018-02
CC1	ST723	SRR3579427		Indonesia - frozen cooked shrimp - 40120
CC1	ST723	SRR1039790		USA - blood - 41211
CC1	ST1	SRR6457825		Ireland - food
CC1	ST723	SRR3985710		Ireland - human - 2011
CC1	ST1	SRR3215346		Italy - cheese
CC1	ST1	SRR3345859		Italy - meat product
CC1	ST1	SRR6475359		Mexico - asadero cheese
CC1	ST1	SRR6304639		Mexico - frozen mamey puree (fruit puree)
CC1	ST1	TS55		Bille & Rocourt 1996 - Bille 1990 Swiss
CC1	ST1017	SRR5764929		Switzerland - meat - 1999
CC1	ST404	SRR1394036		USA - not provided
CC1	ST825	SRR1055854		USA - 41471
CC1	ST1	SRR1745628		USA:RI - green salad
CC1	ST1	SRR6116322		USA:SD - sheep brain (Ovis aries)
CC1	ST1	16SEL568LM	10/9366	Slovenia NRL
CC1	ST1	17SEL32LM	1799	Slovakia NRL
CC1	ST1	14SEL1452LM	2014/8/347	North Macedonia NRL
CC1	ST1	17SEL1LM		France NRL
CC1	ST10	ERS1375051	06CEB122LM	France NRL
CC1	ST328	14SEL873LM		France NRL
CC1	ST1	14SEL397LM		France NRL
CC1	ST1	TS29		Bille & Rocourt 1996 - Linan et al. 1998 USA
CC2	ST2	SRR5115351		Australia - human - 2016
CC2	ST67	SRR5764947		Canada:Alberta - food - 33606
CC2	ST2	SRR5764862		Canada:British Columbia - food - 2009
CC2	ST2	SRR5931807		Chile - not provided
CC2	ST2	SRR3395005		Chile:Araucana - pork pate - 2010
CC2	ST2	ERR2523675		Denmark - not provided - 2010
CC2	ST2	ERR2523768		Denmark - not provided - 2015
CC2	ST2	ERR2523759		Denmark - not provided - 2015
CC2	ST2	SRR1566192		Germany - fetus - 1975
CC2	ST2	ERR2261327		Germany - human listeriosis - 2010
CC2	ST2	SRR3578560		Ireland - human - 2015
CC2	ST2	SRR3215365		Italy - cheese (raw milk) - 2011-05
CC2	ST2	SRR5084553		Italy - environment (cutting board -poultry) - 2005
CC2	ST2	SRR6413642		Japan - squid - 37831
CC2	ST2	SRR5182487		Mexico - cotija cheese - 40436
CC2	ST2	SRR6288283		Mexico - home-made cheese - 37889
CC2	ST2	SRR3173374		Poland - cheese - 39973
CC2	ST2	SRR1610005		South Korea - imitation breaded scallops - 33703
CC2	ST2	SRR1818069		Switzerland - vaucherin cheese - not provided
CC2	ST2	SRR4093253		Ukraine - herrings in oil - 42579
CC2	ST2	SRR3707715		United Kingdom - pate - 1989
CC2	ST2	SRR6807447		United Kingdom:Wales - human - 2011-07
CC2	ST2	SRR4052265		United Kingdom:North of England - human - 2007-01
CC2	ST2	SRR2544687		USA - clinical - 1994
CC2	ST2	SRR1166856		USA - cantaloupe - not provided
CC2	ST2	ERR1100934		not provided
CC2	ST2	ERR1230413		not provided
CC2	ST2	ERR1230410		not provided
CC2	ST2	ERR1738744		France - Food - 2009
CC2	ST2	SRR945155		not provided - blood - 41425
CC2	ST2	SRR1481677		blood - not provided
CC2	ST2	SRR1039785		USA - not provided
CC2	ST2	SRR6956849		USA - food - not provided
CC2	ST2	16SEL786LM	A8	North Macedonia NRL
CC2	ST2	18SEL176LM	DAV170685	Luxembourg NRL
CC2	ST2	17SEL37LM	3676	Slovakia NRL
CC2	ST2	16SEL1179LM	SLCC2020	Austrian NRL
CC2	ST2	16SEL527LM	L637	Slovenian NRL
CC2	ST2	ERS1375112	09CEB339LM	France NRL
CC2	ST2	17SEL376LM		France NRL
CC3	ST66	ERR538102		not provided - 1967
CC3	ST287	ERR1230369		not provided
CC3	ST39	ERR1230363		not provided
CC3	ST117	ERR1230360		not provided

CC3	ST576	ERR1230359		not provided
CC3	ST3	SRR5604250		Chile - frozen cooked mussel meat - 42717
CC3	ST3	SRR3394970		Chile:Los Lagos - bologna sausage - 2010
CC3	ST3	SRR3647048		China - frozen cold smoked salmon snack bites - 42507
CC3	ST3	SRR1982191		China - frozen seafood mix - 42095
CC3	ST3	ERR2512043		Denmark - not provided - 2011
CC3	ST3	SRR1509618		Germany - calf - 1985
CC3	ST3	SRR1509591		Germany - neonate - 1976
CC3	ST3	SRR6457846		Ireland - food - 2009
CC3	ST3	SRR6367670		Israel - vagina - 42944
CC3	ST3	SRR5085098		Italy - roe deer animal - 2008
CC3	ST3	SRR5085108		Italy - chamois animal - 2008
CC3	ST3	SRR5296614		Latvia - fish-latis herring in oil - 41373
CC3	ST3	SRR6425536		Mexico - amber jack fish, frozen - 40745
CC3	ST3	SRR5764934		Switzerland - meat - 1999
CC3	ST3	SRR5764936		Switzerland - sporadic listeriosis case - 2005
CC3	ST3	SRR6784181		Thailand - fish cakes - 38081
CC3	ST3	SRR1640136		United Kingdom - clinical - not provided
CC3	ST3	SRR5159850		United Kingdom - retail meat - not provided
CC3	ST3	SRR5238255		USA - spleen - 2017-01
CC3	ST281	SRR2982078		USA:CA - drain in front of refrigerator - 38587
CC3	ST44	SRR1805444		USA:KS - chicken taquitos - not provided
CC3	ST3	ERS1375013	03EB237LM	France NRL
CC3	ST3	ERS1375115	03EB411LM	France NRL
CC3	ST3	ERS1374965	06CEB216LM	France NRL
CC3	ST3	ERS1375030	10CEB88LM	France NRL
CC3	ST3	ERS1374955	AF160	France NRL
CC4	ST1289	ERR1940776		not provided
CC4	ST397	SRR6784715		USA - not provided
CC4	ST219	SRR6766620		USA - not provided
CC4	ST4	SRR6929849		Netherlands - mushrooms - 43171
CC4	ST55	ERR1230425		not provided
CC4	ST329	ERR1102219		not provided - Food - 2008
CC4	ST317	ERR1102163		not provided - human - 2002
CC4	ST170	ERR2309508		France - clinical sample - 2012
CC4	ST4	SRR3646054		Ireland - human - 2010
CC4	ST4	SRR3578131		Ireland - human - not provided
CC4	ST4	SRR3345852		Italy - milk - 2014-04
CC4	ST4	SRR3345685		Italy - salami - 2013-04
CC4	ST219	SRR6829268		South Africa - not provided
CC4	ST4	SRR5764913		Switzerland - carcasses - 2011
CC4	ST4	SRR5764931		Switzerland - meat - 1999
CC4	ST816	SRR6805284		United Kingdom:London - human - 2015-05
CC4	ST4	SRR6798627		United Kingdom:Midlands and East of England - human - 2015-12
CC4	ST4	SRR974876		USA - amniotic fluid - 41486
CC4	ST4	SRR5117049		USA - ascites fluid - 2016-11
CC4	ST817	SRR1016598		USA - blood - 41534
CC4	ST815	SRR1027067		USA - blood - 41556
CC4	ST783	SRR957725		USA - CSF - 41440
CC4	ST531	SRR3669936		USA - not provided
CC4	ST4	15SEL1281LM		France NRL
CC4	ST4	17SEL381LM	L210	Slovenia NRL
CC4	ST4	17SEL383LM	L230	Slovenia NRL
CC4	ST4	14SEL971LM		France NRL
CC4	ST4	ERS1375029	06CEB271LM	France NRL
CC4	ST4	ERS1375042	08CEB08LM	France NRL
CC4	ST4	ERS1374971	09CEB160LM	France NRL
CC4	ST4	17SEL394LM		France NRL
CC4	ST4	17SEL419LM		France NRL
CC4	ST4	17SEL428LM		France NRL
CC4	ST4	17SEL449LM		France NRL
CC4	ST4	17SEL524LM		France NRL
CC4	ST4	17SEL543LM		France NRL
CC4	ST4	L1031		Slovenia NRL
CC4	ST4	L1350		Slovenia NRL
CC4	ST4	L635		Slovenia NRL
CC5	ST1063	ERR1940723		not provided
CC5	ST820	SRR1048613		not provided - motor frame on cabbage spinner - 41576
CC5	ST5	SRR5115352		Australia - human - 2016
CC5	ST5	SRR1640144		Canada - clinical - not provided
CC5	ST5	SRR5764955		Canada:British Columbia - food processing environment - 2009
CC5	ST5	SRR6783012		Chile - frozen cooked mussel meat - 38325
CC5	ST5	SRR3394987		Chile:Valparaiso - cerebrospinal fluid - 2010
CC5	ST5	ERR2512032		Denmark - not provided - 2010
CC5	ST5	ERR2512033		Denmark - not provided - 2011
CC5	ST5	ERR2261317		Germany - human listeriosis - 2008
CC5	ST5	ERR2261329		Germany - human listeriosis - 2010
CC5	ST5	SRR6457795		Ireland - - 2012
CC5	ST5	SRR6457847		Ireland - food - 2010
CC5	ST5	SRR5087526		Italy - blood - 2015
CC5	ST5	SRR5085209		Italy - blood - 2016
CC5	ST5	SRR5061761		Italy - swine sausage - 2003
CC5	ST5	SRR5896238		Mexico - frozen burrito - 42803
CC5	ST745	SRR1805409		chicken, pork, beef franks - not provided
CC5	ST1178	SRR1805396		not provided - smoked ham slice - 36441
CC5	ST5	SRR1373597		Puerto Rico - ham steak - 2013

CC5	ST5	SRR6829224		South Africa - not provided
CC5	ST5	SRR3453147		South Korea - frozen snow cooked crabmeat - 42324
CC5	ST5	SRR1818073		Spain - chorizos (pork sausages) - not provided
CC5	ST5	SRR5764978		Switzerland - food processing environment - 2011
CC5	ST5	SRR5318944		Ukraine - fish-herring fillet in oil - 40904
CC5	ST5	SRR5344713		United Kingdom - retail meat - not provided
CC5	ST5	SRR4052285		United Kingdom:London - human - 2013-03
CC5	ST5	SRR4052213		United Kingdom:Midlands and East of England - human - 2014-08
CC5	ST784	SRR1001047		USA - blood and CSF, same patterns - 41506
CC5	ST5	SRR1575073		USA - brain abscess - 2014-08
CC5	ST5	SRR2048383		USA - Cantaloupe - not provided
CC5	ST5	SRR2047399		USA - Dairy_Ice cream - 42048
CC5	ST820	SRR2046982		USA - Environmental-Inside of floor drain by cabbage flume - 41576
CC5	ST784	SRR6424894		USA - not provided
CC5	ST879	SRR974864		USA - not provided
CC5	ST819	SRR3181832		USA:MN - swab - 40858
CC5	ST1093	SRR2969589		USA:FL - ham salad - 42082
CC5	ST1094	SRR3082370		USA:GA - Environmental - 42018
CC5	ST1106	SRR5202192		USA:NE - Environmental:non-food-contact surface - 2016
CC5	ST1063	SRR4305576		USA:PA - Environmental:non-food-contact surface - 2013
CC5	ST1095	SRR1463370		USA:PA - environmental:non-food-contact surface - 2013
CC5	ST5	ERS1375016	06CEB107LM	France NRL
CC5	ST5	12CEB320LM		France NRL
CC5	ST5	16SEL1277LM		USA Cornell
CC5	ST5	16SEL518LM	L567	Slovenia NRL
CC5	ST5	16SEL667LM	12/25731	Slovenia NRL
CC5	ST5	17SEL13LM		France NRL
CC5	ST5	17SEL456LM		France NRL
CC5	ST5	18SEL183LM	F57E113	Luxembourg NRL
CC5	ST5	18SEL184LM	DAV180122	Luxembourg NRL
CC6	ST615	ERR1100954		not provided
CC6	ST179	ERR2523711		Denmark - not provided - 2011
CC6	ST6	ERR2512034		Denmark - not provided - 2011
CC6	ST6	ERR2261558		Germany - human listeriosis - 2016
CC6	ST6	ERR2261725		Germany - human listeriosis - 2017
CC6	ST6	SRR6457783		Ireland - food - 2013
CC6	ST6	SRR3578559		Ireland - human - 2015
CC6	ST6	SRR5085117		Italy - bovine Stool - 2011
CC6	ST6	SRR3345560		Italy - food(ready-to-eat) - 2013-02
CC6	ST6	SRR5861482		Lithuania - Roach dry eviscerated salted fish - 42901
CC6	ST1292	SRR5632023		Mexico - avocado - 41835
CC6	ST6	SRR3229330		Philippines - Grated Coconut - 42424
CC6	ST6	SRR7056253		South Africa - blood culture - 43016
CC6	ST6	SRR7056256		South Africa - food - 43112
CC6	ST6	SRR5764979		Switzerland - carcasses - 2011
CC6	ST6	SRR5160282		United Kingdom - retail meat - not provided
CC6	ST6	SRR7277883		United Kingdom:United Kingdom - human - 2018-05
CC6	ST6	SRR3707892		USA - deli meat - 2002
CC6	ST823	SRR1005723		USA - pleural fluid - 41505
CC6	ST6	SRR1269966		USA - retail deli - 40282
CC6	ST1101	SRR1220701		USA:AR - lettuce - 2012-05
CC6	ST1292	SRR1578489		USA:MN - avocado - 41835
CC6	ST215	SRR5498107		USA:TX - environmental:non-food-contact surface - 2012
CC6	ST6	ERS1374984	06CEB188LM	France NRL
CC6	ST6	ERS1375003	06CEB211LM	France NRL
CC6	ST6	ERS1375125	06CEB542LM	France NRL
CC6	ST6	17SEL30LM	1118	Slovakia NRL
CC6	ST6	17SEL385LM	L351	Slovakia NRL
CC6	ST6	16SEL572LM	10/11192	Slovenia NRL
CC6	ST6	16SEL573LM	10/14041	Slovenia NRL
CC6	ST6	17SEL497LM		France NRL
CC6	ST6	17SEL591LM		France NRL
CC6	ST6	17SEL82LM		France NRL
CC6	ST6	17SEL460LM		France NRL
CC7	ST1300	ERR1940750		not provided
CC7	ST830	SRR1201367		not provided
CC7	ST12	ERR538099		not provided - 1924
CC7	ST98	ERR538107		not provided - 1937
CC7	ST107	ERR538098		not provided
CC7	ST85	SRR3099265		not provided
CC7	ST624	ERR1100914		not provided
CC7	ST519	ERR1230448		not provided
CC7	ST7	SRR5115349		Australia - human - 2016
CC7	ST7	SRR2156619		Canada - salmon, frozen, smoked - 40613
CC7	ST7	SRR5764991		Canada:Alberta - food - 1990
CC7	ST7	SRR1509627		Chile - fresh hass avocados - 41786
CC7	ST7	SRR3394984		Chile:Santiago - blood - 2010
CC7	ST7	SRR3242137		Colombia - avocado pulp - 2016
CC7	ST7	ERR2523685		Denmark - not provided - 2010
CC7	ST7	ERR2523757		Denmark - not provided - 2015
CC7	ST7	ERR2261704		Germany - human listeriosis - 2016
CC7	ST691	ERR2261655		Germany - human listeriosis - 2016
CC7	ST7	ERR2261712		Germany - human listeriosis - 2017
CC7	ST7	SRR6457877		Ireland - environmental - 2012
CC7	ST7	SRR6457868		Ireland - food - 2011
CC7	ST7	SRR5085099		Italy - bovine Stool - 2011

CC7	ST7	SRR3215356		Italy - rice - 2011-01
CC7	ST7	SRR1946888		Mexico - avocado pulp - 37826
CC7	ST7	SRR1535729		Mexico - avocados - 41794
CC7	ST561	SRR1041631		not provided - blood - 41458
CC7	ST7	SRR3674657		Netherlands - smoked salmon red sockeye - 42482
CC7	ST12	SRR1816401		not collected - not provided
CC7	ST158	SRR6958703		United Kingdom - not provided
CC7	ST107	SRR1685979		unknown - unknown - not provided
CC7	ST561	SRR1039784		USA - - not provided
CC7	ST788	SRR1779454		USA - blood - 2014-12
CC7	ST877	SRR3991321		USA - blood - 2016-07
CC7	ST561	SRR2048384		USA - Cantaloupe - not provided
CC7	ST1399	SRR6116309		USA:SD - bovine brain stem (Bos taurus) - 2017
CC7	ST7	SRR3215115		USA:SD - Environmental:food-contact surface - 2016
CC7	ST111	16SEL1320LM	SLCC 203	Austria NRL
CC7	ST7	16SEL1401LM	VI-58126	Norway NRL
CC7	ST7	16SEL17LM		France NRL
CC7	ST691	16SEL563LM	L538	Slovenia NRL
CC7	ST624	15SEL842LM		France NRL
CC7	ST511	18SEL182LM	DAV170094	Luxembourg NRL
CC7	ST7	CEB454LM		France NRL
CC7	ST7	17SEL36LM	3337	Slovakia NRL
CC7	ST7	17SEL15LM		France NRL
CC7	ST7	17SEL401LM		France NRL
CC7	ST7	17SEL102LM		France NRL
CC8	ST16	SRR2133394		USA:TX - environmental swab - 42163
CC8	ST1294	ERR1940711		not provided
CC8	ST1295	ERR1940712		not provided
CC8	ST1296	ERR1940716		not provided
CC8	ST742	ERR1940717		not provided
CC8	ST1294	ERR1940720		not provided
CC8	ST1297	ERR1940789		not provided
CC8	ST1298	ERR1940793		not provided
CC8	ST743	ERR1947066		not provided
CC8	ST120	SRR1520059		USA - blood - 2014-06
CC8	ST551	SRR1174766		USA - Bile fluid - 41655
CC8	ST17	ERR1599684		not provided - 2003
CC8	ST292	SRR3745084		Canada - <not provided> - not provided
CC8	ST120	SRR5167786		Canada - blood - 2008
CC8	ST1022	SRR5764960		Canada:Alberta - food - 32904
CC8	ST1025	SRR5764849		Canada:Alberta - food - 33100
CC8	ST8	SRR6413646		Chile - cold smoked salmon - 41256
CC8	ST8	SRR3394985		Chile:Los Lagos - cerebrospinal fluid - 2011
CC8	ST8	SRR3606577		China - breaded crab claws with real pincer - 39372
CC8	ST8	SRR2050964		China - crab cakes - 2015
CC8	ST8	ERR2523678		Denmark - not provided - 2010
CC8	ST8	ERR2523714		Denmark - not provided - 2011
CC8	ST8	ERR2261316		Germany - human listeriosis - 2008
CC8	ST551	ERR2261323		Germany - human listeriosis - 2010
CC8	ST8	ERR2261344		Germany - human listeriosis - 2012
CC8	ST8	SRR6457790		Ireland - 2012
CC8	ST8	SRR6457858		Ireland - environmental - 2011
CC8	ST8	SRR3345539		Italy - cheese - 2012-08
CC8	ST8	SRR3345810		Italy - chicken - 2013-06
CC8	ST8	SRR3099225		Norway - poultry processing facility - 2013
CC8	ST8	SRR3099222		Norway - salmon processing facility - 2001
CC8	ST8	SRR975366		Poland - salmon, graved, norwegian - 39706
CC8	ST8	SRR6829223		South Africa - not provided
CC8	ST8	SRR1605033		United Kingdom - retail meat - 2006-2008
CC8	ST8	SRR2831326		USA:CA - frozen cherries - 2015
CC8	ST8	17SEL426LM		France NRL
CC8	ST16	17SEL59LM		France NRL
CC8	ST8	17SEL6LM		France NRL
CC8	ST8	17SEL43LM	7161	Slovakia NRL
CC8	ST8	17SEL45LM	11037	Slovakia NRL
CC8	ST8	ERS1375032	09CEB19LM	France NRL
CC8	ST8	ERS1374957	10CEB335LM	France NRL
CC8	ST8	ERS1374975	11CEB391LM	France NRL
CC8	ST8	17SEL12LM		France NRL
CC9	ST751	ERR1947094		not provided
CC9	ST713	ERR1599707		not provided - 2005
CC9	ST122	ERR538101		not provided - 1935
CC9	ST441	ERR1100969		not provided
CC9	ST356	ERR1230468		not provided
CC9	ST9	SRR1656965		Austria - bacon pastry ("verhackert") - 2014
CC9	ST9	SRR1656979		Austria - bovine meat - 2014
CC9	ST35	SRR1665052		Canada - Animal - not provided
CC9	ST9	SRR6321577		Canada - cooked whole crab - 37586
CC9	ST9	SRR5764875		Canada:Alberta - food - 33235
CC9	ST9	SRR5931811		Chile - not provided
CC9	ST9	ERR2523744		Denmark - not provided - 2012
CC9	ST580	ERR2523756		Denmark - not provided - 2015
CC9	ST9	ERR2523751		Denmark - not provided - 2015
CC9	ST9	SRR1656981		Germany - cheese - 2014
CC9	ST9	SRR1520049		Germany - chicken - not provided
CC9	ST9	SRR6457867		Ireland - food - 2011

CC9	ST9	SRR6958565		Israel - food - not provided
CC9	ST9	SRR5912800		Italy - bacon - 2012-04
CC9	ST9	SRR5087866		Italy - blood - 2014
CC9	ST9	SRR6806305		Korea:Republic Of (South) - pollack roe, seasoned - 39842
CC9	ST9	SRR6413628		Korea:Republic Of (South) - snow crab - 37784
CC9	ST9	SRR5296611		Latvia - fish-latis herring in oil - 41382
CC9	ST9	SRR5318935		Latvia - fish-latis herring in oil - 41382
CC9	ST622	SRR5344726		United Kingdom - retail meat - not provided
CC9	ST9	SRR5344716		United Kingdom - retail meat - not provided
CC9	ST1113	SRR3632211		USA - Cantaloupe chunks - 42501
CC9	ST9	SRR1597491		USA - CSF - 41915
CC9	ST9	SRR2583989		USA:FL - deli turkey - 39923
CC9	ST510	SRR3095285		USA:IA - Environmental:non-food-contact surface - 2007
CC9	ST115	SRR5497383		USA:NC - environmental:non-food-contact surface - 2012
CC9	ST9	15SEL1695LM	12/448	Irland NRL
CC9	ST9	ERS1374986	03EB210LM	France NRL
CC9	ST9	ERS1375102	05CEB303LM	France NRL
CC9	ST9	15SEL1533LM	BfR-Ll-00160	German NRL
CC9	ST9	16SEL1407LM	VI-58155	Norway NRL
CC9	ST9	16SEL863LM	57592	Norway NRL
CC9	ST1116	15SEL699LM		IFIP
CC9	ST497	16SEL1197LM	SLCC3860	Austria NRL
CC9	ST356	16SEL1169LM	SLCC762	Austria NRL
CC9	ST622	17SEL375LM		France NRL
CC9	ST580	17SEL494LM		France NRL
CC9	ST580	17SEL41LM	7059	Slovakia NRL
CC9	ST9	17SEL44LM	9870	Slovakia NRL
CC11	ST451	GCF_001709825		Canada
CC11	ST451	GCF_001710425		Canada
CC11	ST451	GCF_001761015		USA
CC11	ST451	GCF_002443615		USA
CC11	ST451	GCF_003189325		Canada
CC11	ST451	GCF_003189405		Canada
CC11	ST451	GCF_003588125		UK
CC11	ST451	GCF_003589045		UK
CC11	ST451	GCF_003606615		USA
CC11	ST451	GCF_003606625		USA
CC11	ST451	L295		Slovenia NRL
CC11	ST451	L296		Slovenia NRL
CC11	ST451	L297		Slovenia NRL
CC11	ST451	L300		Slovenia NRL
CC11	ST451	ERS1375007	12CEB24LM	France NRL
CC11	ST451	17SEL571LM		France NRL
CC11	ST451	18SEL139LM		France NRL
CC11	ST451	18SEL581LM		France NRL
CC11	ST451	19SEL004LM		France NRL
CC14	ST1314	ERR1940726		not provided
CC14	ST399	ERR1817011		not provided - Factory - 2009
CC14	ST14	SRR1982200		Chile - frozen salmon mince grilled - 40461
CC14	ST399	ERR2523713		Denmark - not provided - 2011
CC14	ST14	ERR2512037		Denmark - not provided - 2011
CC14	ST206	ERR1738662		France - Food - 2001
CC14	ST14	ERR2261322		Germany - human listeriosis - 2010
CC14	ST399	ERR2261580		Germany - human listeriosis - 2016
CC14	ST14	ERR2261603		Germany - human listeriosis - 2016
CC14	ST14	SRR3646063		Ireland - human - 2011
CC14	ST14	SRR5088121		Italy - blood - 2014
CC14	ST14	SRR5087868		Italy - blood/CSF - 2014
CC14	ST14	SRR5087869		Italy - CSF - 2014
CC14	ST206	SRR3345877		Italy - Meat (kebob) - 2012-12
CC14	ST14	SRR5087838		Italy - not provided - 2013
CC14	ST14	SRR5160241		United Kingdom - retail meat - not provided
CC14	ST14	SRR4052199		United Kingdom:North of England - human - 2014-01
CC14	ST14	SRR7180013		United Kingdom - human - 2016-07
CC14	ST206	SRR7179991		United Kingdom - human - 2017-04
CC14	ST14	GCF_003588985		UK
CC14	ST14	GCF_003606555		USA
CC14	ST14	16SEL1295LM		France NRL
CC14	ST14	16SEL1371LM	15	North Macedonia NRL
CC14	ST14	16SEL1400LM	VI-58124	Norway NRL
CC14	ST14	16SEL1404LM	VI-58142	Norway NRL
CC14	ST14	18SEL159LM		France NRL
CC14	ST14	18SEL160LM		France NRL
CC14	ST14	18SEL175LM	DAV170122	Luxembourg NRL
CC14	ST14	18SEL25LM		France NRL
CC14	ST14	11CEB642LM		France NRL
CC14	ST91	ERR1738742		France - Food - 2009
CC14	ST360	SRR1640172		unknown - animal - not provided
CC14	ST91	ERR1947108		not provided
CC14	ST91	SRR4052169		United Kingdom:South of England - human - 2009-08
CC14	ST91	SRR5765001		Canada:British Columbia - food - 2009
CC14	ST360	SRR2777519		Canada:Ontario - Food - 2010
CC14	ST91	SRR5085103		Italy - bovine Stool - 2010
CC14	ST91	SRR3345843		Italy - poultry meat - 2014-11
CC14	ST91	14SEL840LM		France NRL
CC14	ST91	ERS1375018	09CEB335LM	France NRL

CC14	ST91	16SEL664LM	12/20566	Slovenia NRL
CC14	ST360	16SEL1276LM		USA Cornell
CC14	ST91	RL15000511		Denmark NRL - Painset et al. 2019
CC14	ST91	RL15000441		Slovakia NRL - Painset et al. 2019
CC14	ST91	RL15000096		Poland NRL - Painset et al. 2019
CC14	ST91	GCF_003588155		UK
CC14	ST360	GCF_003187985		Canada:Ontario
CC14	ST91	GCF_003097415		USA
CC14	ST91	GCF_002488205		USA
CC14	ST91	GCF_002488035		USA
CC14	ST91	GCF_002445485		USA
CC14	ST91	GCF_001760305		USA
CC14	ST91	GCF_001711255		Canada:British Columbia
CC14	ST91	GCF_001583285		USA
CC14	ST91	GCF_000306985		Austria
CC18	ST481	ERR664779		not provided
CC18	ST18	ERR2523719		Denmark - not provided - 2011
CC18	ST18	ERR2309482		France - clinical sample - 2011
CC18	ST18	ERR1738750		France - Food - 2009
CC18	ST18	ERR2261335		Germany - human listeriosis - 2011
CC18	ST18	SRR3436432		Ireland - human - 2013
CC18	ST18	SRR5087479		Italy - blood - 2015
CC18	ST18	SRR5087555		Italy - blood - 2015
CC18	ST18	SRR3345864		Italy - minced meat - 2014-10
CC18	ST18	SRR5088141		Italy - not provided - 2014
CC18	ST18	SRR5160278		United Kingdom - retail meat - not provided
CC18	ST18	SRR1789032		USA - blood - 41974
CC18	ST18	SRR1996258		USA - cerebrospinal fluid - 2015-02
CC18	ST18	SRR3175185		USA:FL - smoked salmon - 39602
CC18	ST18	SRR5133474		USA:NY - sprats in spicy brine - 41473
CC18	ST18	ERS1375087	09CEB582LM	France NRL
CC18	ST18	17SEL3LM		France NRL
CC18	ST18	17SEL465LM		France NRL
CC18	ST18	17SEL4LM		France NRL
CC18	ST18	17SEL574LM		France NRL
CC18	ST18	17SEL575LM		France NRL
CC18	ST18	17SEL72LM		France NRL
CC18	ST18	18SEL138LM		France NRL
CC18	ST18	L562		Slovenia NRL
CC18	ST18	L563		Slovenia NRL
CC18	ST18	L564		Slovenia NRL
CC19	ST398	SK-OVI-CP-23		Slovakia NRL
CC19	ST398	L1053		Slovenia NRL
CC19	ST398	GCF_900169945		Italy
CC19	ST398	GCF_003587725		UK
CC19	ST398	GCF_002446115		USA
CC19	ST398	GCF_002445745		USA
CC19	ST398	GCF_002445365		USA
CC19	ST398	GCF_002444525		USA
CC19	ST398	GCF_002443915		USA
CC19	ST398	GCF_002443595		USA
CC19	ST398	GCF_000613165		Austria
CC19	ST398	ADN_NRL_HU_332		Hungary NRL
CC19	ST398	ADN_NRL_HU_232		Hungary NRL
CC19	ST398	ADN_NRL_HU_208		Hungary NRL
CC19	ST398	ADN_NRL_HU_164		Hungary NRL
CC19	ST398	23EA15SEL663LM		France NRL
CC19	ST398	23EA15SEL662LM		France NRL
CC19	ST398	23EA15SEL661LM		France NRL
CC20	ST1319	ERR1947075		not provided
CC20	ST20	SRR5764905		Canada:Alberta - food - 33242
CC20	ST20	SRR5764902		Canada:Alberta - food - 33242
CC20	ST647	ERR1738823		France - Food - 1992
CC20	ST20	ERR1738732		France - Food - 2008
CC20	ST20	ERR1738737		France - Food - 2009
CC20	ST20	SRR6457875		Ireland - food - 2012
CC20	ST20	SRR3578558		Ireland - human - 2015
CC20	ST20	SRR3399273		Ireland - human - 2015
CC20	ST20	SRR3345848		Italy - milk - 2014-02
CC20	ST20	SRR5764914		Switzerland - carcasses - 2011
CC20	ST20	SRR6798519		United Kingdom:London - human - 2013-04
CC20	ST20	SRR6798580		United Kingdom:Midlands and East of England - human - 2015-10
CC20	ST20	SRR6798553		United Kingdom:None - human - 2011-11
CC20	ST424	SRR1695781		USA - blood - 2014-10
CC20	ST20	SRR5998769		USA - blood - 2017-08
CC20	ST647	SRR1506612		USA - blood - 41798
CC20	ST424	SRR1411154		USA:CT - beef blend dog food - 41781
CC20	ST20	SRR3928666		USA:FL - smoked salmon - 38547
CC20	ST20	SRR4473727		USA:IA - Environmental sponge - 42634
CC20	ST20	SRR7253631		USA:IA - Surface, floors and Drains - 43158
CC20	ST20	SRR5104737		USA:OH - spinach - 2012
CC20	ST20	SRR1451245		USA:WI - Environmental isolate, sponge swab - 41740
CC20	ST20	ERS1375114	08CEB286LM	France NRL
CC20	ST20	ERS1375035	09CEB147LM	France NRL
CC20	ST20	17SEL73LM		France NRL
CC20	ST20	17SEL74LM		France NRL

CC20	ST20	18SEL136LM		France NRL
CC20	ST647	ERS1374958	BO2	France NRL
CC21	ST336	ERR1940785		not provided
CC21	ST21	ERR1940756		not provided
CC21	ST1302	ERR1940779		not provided
CC21	ST725	ERR1940739		not provided
CC21	ST21	ERR2523742		Denmark - not provided -
CC21	ST21	ERR1738674		France - food -
CC21	ST21	ERR2261681		Germany - human listeriosis -
CC21	ST21	SRR5085102		Italy - bovine stool - Dept. Vet. Sciences, UNITO
CC21	ST21	SRR1395302		Mexico - organic red mango - FDA
CC21	ST21	SRR5159837		United Kingdom - retail meat - FERA
CC21	ST21	SRR6798607		United Kingdom:Midlands and East of England - human - PHE
CC21	ST21	SRR1597475		USA - blood - CDC
CC21	ST21	SRR3089956		USA - cerebral spinal fluid - CDC
CC21	ST21	SRR6283485		USA - not provided
CC21	ST21	SRR6413635		USA:WA - smoked salmon - FDA
CC21	ST21	SRR5280368		USA:WA - swab - conveyer belt - FDA
CC21	ST21	SRR2915944		USA:AK - raw milk - AK
CC21	ST21	SRR2184256		USA:CA - Environmental:non-food-contact surface - USDA-FSIS
CC21	ST21	SRR3372265		USA:NY - trough water - USDA-ARS
CC21	ST21	17SEL88LM		France NRL
CC26	ST26	ERR2523789		Denmark - not provided - 2015
CC26	ST26	ERR2486989		France - Dairy products - 2007
CC26	ST26	ERR1738839		France - Food - 1989
CC26	ST26	ERR1738749		France - Food - 2009
CC26	ST26	ERR1738741		France - Food - 2009
CC26	ST26	SRR1535739		France - roquefort vieux berger - 41808
CC26	ST26	SRR3168976		Italy - swine salami - 2003
CC26	ST26	SRR5061780		Italy - swine salami - 2003
CC26	ST26	SRR5337912		United Kingdom:None - human - 2015-12
CC26	ST26	SRR7164132		United Kingdom:United Kingdom - human - 2016-01
CC26	ST26	SRR7167759		United Kingdom:United Kingdom - human - 2016-01
CC26	ST26	SRR4032186		USA - blood - 2016-07
CC26	ST26	SRR1575063		USA - blood - 41897
CC26	ST26	SRR3147938		USA - not provided
CC26	ST26	SRR1805496		USA:MD - classic chicken salad - not provided
CC26	ST26	SRR2960805		USA:WI - food - 42296
CC26	ST26	ERS1375044	09CEB334LM	France NRL
CC26	ST26	L719		Slovenia NRL
CC26	ST26	L722		Slovenia NRL
CC26	ST26	L727		Slovenia NRL
CC26	ST26	L733		Slovenia NRL
CC26	ST26	ERS1375088	SO190	France NRL
CC26	ST26	ERS1374959	09CEB411LM	France NRL
CC29	ST586	ERR1940770		not provided
CC29	ST29	ERR2523715		Denmark - not provided - 2011
CC29	ST29	SRR3391877		Italy - cheese - 2003
CC29	ST427	SRR3345687		Italy - chicken - 2013-04
CC29	ST427	SRR5084593		Italy - swine meat - 2005
CC29	ST29	SRR3391847		Italy - swine sausage - 2004
CC29	ST29	SRR7180011		United Kingdom:United Kingdom - human - 2018-04
CC29	ST29	SRR1263975		USA - bone - 41726
CC29	ST29	SRR2048382		USA - cantaloupe - not provided
CC29	ST29	SRR1575076		USA - not provided
CC29	ST849	SRR1910314		USA - not provided
CC29	ST29	SRR3278102		USA:MI - frozen blueberries - 40339
CC29	ST29	SRR5417947		USA:CA - environmental swab - 2017
CC29	ST1123	SRR1812798		USA:NH - aged raw milk cheese prep 11/6/14 - 42016
CC29	ST29	17SEL433LM		France NRL
CC29	ST29	17SEL50LM		France NRL
CC29	ST29	17SEL568LM		France NRL
CC29	ST29	17SEL71LM		France NRL
CC29	ST29	L1231		Slovenia NRL
CC29	ST29	L1235		Slovenia NRL
CC29	ST29	17SEL70LM		France NRL
CC31	ST748	ERR1947107		not provided
CC31	ST325	ERR1947109		not provided
CC31	ST325	SRR3945604		France - cabrioulet cheese (aged over 2 months) - 41796
CC31	ST31	ERR1738842		France - Food - 1989
CC31	ST31	SRR6457856		Ireland - environmental - 2011
CC31	ST325	SRR5084469		Italy - bovine cheese - blue cheese - 2004
CC31	ST325	SRR3345846		Italy - swab (dairy plant) - 2014-02
CC31	ST31	SRR5047489		Japan - dried sailfin sandfish - 42655
CC31	ST325	SRR7167741		United Kingdom:United Kingdom - human - 2017-09
CC31	ST1425	SRR6425173		USA - chicken with organic vegetables meals for dogs - 43076
CC31	ST31	SRR3611559		USA:AL - Environmental:non-food-contact surface - 2016
CC31	ST325	SRR1818017		USA:CA - blue-veined, mold-ripened cheese - 2001
CC31	ST31	SRR1744061		USA:CA - environmental swab - 41997
CC31	ST1177	SRR1818015		USA:LA - king creole cubed andouille - not provided
CC31	ST31	SRR3710278		USA:MO - Environmental:non-food-contact surface - 2016
CC31	ST31	ERS1374961	05CEB474LM	France NRL
CC31	ST31	ERS1374950	06CEB204LM	France NRL
CC31	ST31	ERS1375037	06CEB205LM	France NRL
CC31	ST31	ERS1375122	11CEB350LM	France NRL
CC31	ST31	ERS1375043	11CEB354LM	France NRL

CC31	ST31	ERS1375017	11CEB363LM	France NRL
CC31	ST31	ERS1375026	11CEB371LM	France NRL
CC31	ST31	ERS1375058	11CEB446LM	France NRL
CC31	ST31	ERS1375055	12CEB28LM	France NRL
CC31	ST31	ERS1374968	AF114	France NRL
CC31	ST31	ERS1375022	BO18	France NRL
CC31	ST31	ERS1375072	BO38	France NRL
CC31	ST31	ERS1374939	SO95	France NRL
CC31	ST31	17SEL370LM		France NRL
CC37	ST728	ERR1947079		not provided
CC37	ST747	ERR1947101		not provided
CC37	ST37	SRR1640175		Canada - clinical - not provided
CC37	ST37	SRR5764867		Canada:Nova Scotia - water - 41037
CC37	ST37	SRR1378345		Chile - avocado - 41774
CC37	ST37	ERR2523773		Denmark - not provided - 2015
CC37	ST37	ERR1738813		France - Food - 2001
CC37	ST37	ERR2261685		Germany - human listeriosis - 2016
CC37	ST37	SRR6457787		Ireland - food - 2013
CC37	ST37	SRR3646064		Ireland - human - 2011
CC37	ST37	SRR5087876		Italy - blood - 2014
CC37	ST37	SRR3345678		Italy - meat - 2013-03
CC37	ST37	SRR5318937		Latvia - fish-latis herring in oil - 41373
CC37	ST37	SRR3278088		Mexico - frozen mild guacamole - 40437
CC37	ST37	SRR7239888		Netherlands - not provided - 43171
CC37	ST37	ERR2495156		Norway - cheese - 2018
CC37	ST37	SRR5159843		United Kingdom - retail meat - not provided
CC37	ST37	SRR6798573		United Kingdom:London - human - 2015-10
CC37	ST37	SRR6808578		United Kingdom:Midlands and East of England - human - 2011-12
CC37	ST197	SRR2915355		USA - blood - 2015-10
CC37	ST37	SRR5805550		USA:CO - green chile Ingredient - not provided
CC37	ST197	SRR6211450		USA:FL - avocado salad - 43013
CC37	ST643	SRR2848348		USA:MI - cantaloupe - 41513
CC37	ST37	SRR3571296		USA:MI - milk - 2007
CC37	ST37	16SEL709LM	14/12133	Slovenia NRL
CC37	ST37	16SEL718LM	14/19585	Slovenia NRL
CC37	ST37	17SEL103LM		France NRL
CC37	ST37	17SEL105LM		France NRL
CC37	ST37	17SEL441LM		France NRL
CC37	ST37	17SEL443LM		France NRL
CC37	ST37	17SEL463LM		France NRL
CC37	ST37	17SEL522LM		France NRL
CC37	ST37	18SEL147LM		France NRL
CC37	ST37	18SEL180LM	DAV160999	Luxembourg NRL
CC37	ST37	18SEL181LM	DAV170395	Luxembourg NRL
CC37	ST37	ERS1375086	AF143	France NRL
CC54	ST54	07CEB374LM		France NRL
CC54	ST54	16SEL579LM		France NRL
CC54	ST54	16SEL643LM		France NRL
CC54	ST54	16SEL686LM		France NRL
CC54	ST54	19SEL854LM		France NRL
CC54	ST54	19SEL865LM		France NRL
CC54	ST54	19SEL880LM		France NRL
CC54	ST54	19SEL881LM		France NRL
CC54	ST54	20SEL259LM		France NRL
CC54	ST54	20SEL260LM		France NRL
CC54	ST54	20SEL292LM		France NRL
CC54	ST54	20SEL302LM		France NRL
CC54	ST54	21SEL8LM		France NRL
CC54	ST54	21SEL14LM		France NRL
CC54	ST54	GCF_001564955		France NRL
CC54	ST54	GCF_002028125		Ireland
CC54	ST54	GCF_002029185		Ireland
CC54	ST54	GCF_002444305		USA
CC54	ST54	GCF_002445005		USA
CC54	ST54	GCF_002912245		Ireland
CC54	ST54	GCF_003588205		UK
CC54	ST54	RL15000399		France NRL
CC54	ST54	RL15000409		France NRL
CC59	ST1316	ERR1947062		not provided
CC59	ST59	SRR6966186		Argentina - frozen shell-on shrimp - 37271
CC59	ST59	SRR1640123		Canada - food - 2009
CC59	ST59	ERR2523673		Denmark - not provided - 2010
CC59	ST59	ERR2523709		Denmark - not provided - 2011
CC59	ST59	ERR2309461		France - clinical sample - 2010
CC59	ST59	ERR1738707		France - food - 2006
CC59	ST59	SRR3985711		Ireland - human - 2012
CC59	ST59	SRR3391838		Italy - meat - 2004
CC59	ST59	SRR3345927		Italy - smoked salmon - 2011-08
CC59	ST59	SRR1610008		Italy - vol au vent shrimp - 1993-06
CC59	ST59	SRR6926203		Korea:Republic Of (South) - cooked snow crab - 40751
CC59	ST59	SRR5764911		Switzerland - carcasses - 2011
CC59	ST59	SRR1605030		United Kingdom - retail meat - 2006-2008
CC59	ST59	SRR6807436		United Kingdom:North of England - human - 2011-10
CC59	ST59	SRR1535715		USA:CA - hass avocados - 41787
CC59	ST59	SRR5875059		USA:CA - swab - 40766
CC59	ST59	SRR6490545		USA:CA - tuna saku - 43110

CC59	ST59	SRR1411140		USA:CT - beef blend dog food - 41781
CC59	ST59	SRR3198660		USA:MN - milk - 2014
CC59	ST59	SRR5133496		USA:NY - herring filet in oil - 41464
CC59	ST59	SRR3223769		USA:OH - milk filter - 2007
CC59	ST59	SRR5422022		USA:TX - avocado - 41844
CC59	ST59	ERS1375031	06CEB443LM	France NRL
CC59	ST59	ERS1374960	07CEB456LM	France NRL
CC59	ST59	ERS1375014	11CEB633LM	France NRL
CC59	ST59	16SEL638LM	11/21955	Slovenia NRL
CC59	ST59	16SEL692LM	13/12752	Slovenia NRL
CC59	ST59	17SEL585LM		France NRL
CC59	ST59	17SEL517LM		France NRL
CC77	ST77	ERR2523781		Denmark - not provided - 2015
CC77	ST77	SRR7204448		United Kingdom:United Kingdom - human - 2016-01
CC77	ST77	SRR7172534		United Kingdom:United Kingdom - human - 2016-01
CC77	ST77	SRR7172408		United Kingdom:United Kingdom - human - 2016-06
CC77	ST77	SRR5861480		USA:IL - spongesicles - 42878
CC77	ST77	16SEL567LM	10/7646	Slovenia NRL
CC77	ST77	16SEL576LM	10/18625	Slovenia NRL
CC77	ST77	16SEL589LM	11/7542	Slovenia NRL
CC77	ST77	16SEL639LM	11/25198	Slovenia NRL
CC77	ST77	18SEL185LM	DAV170428	Luxembourg NRL
CC77	ST77	21EA15SEL1187LM		France NRL
CC77	ST77	21EA15SEL1197LM		France NRL
CC77	ST77	21EA16SEL208LM		France NRL
CC77	ST77	21EA16SEL216LM		France NRL
CC77	ST77	21EA16SEL254LM		France NRL
CC77	ST77	21EF15SEL1172LM		France NRL
CC77	ST77	21NA08CEB284LM		France NRL
CC77	ST77	21NA08CEB319LM		France NRL
CC77	ST77	21NA09CEB565LM		France NRL
CC77	ST77	21NA09CEB593LM		France NRL
CC77	ST77	21NA12CEB1187LM		France NRL
CC77	ST77	21NA12CEB856LM		France NRL
CC77	ST77	21NA16SEL576LM		France NRL
CC77	ST77	21NA16SEL589LM		France NRL
CC77	ST77	21NAIE22		France NRL
CC77	ST77	21NAIE45		France NRL
CC77	ST77	16SEL639LM		France NRL
CC87	ST87	SRR6366470		Canada - tuna tataki - FDA
CC87	ST87	SRR3747655		China - cold smoked Atlantic salmon - FDA
CC87	ST846	SRR975365		China - crawfish, frozen, cooked - FDA
CC87	ST87	SRR3647046		China - frozen cold smoked salmon snack bites - FDA
CC87	ST87	SRR5088138		Italy - not provided - Department of Health Sciences, UNIMI
CC87	ST847	SRR975360		Mexico - frozen guacamole - FDA
CC87	ST87	SRR3945587		Portugal - sheep's milk cheese - FDA
CC87	ST87	SRR4052133		United Kingdom:London - human - PHE
CC87	ST87	SRR4052050		United Kingdom:Midlands and East of England - human - PHE
CC87	ST87	SRR6369102		USA:CA - environmental swab - CDPH FDLB
CC87	ST847	SRR6178849		USA:FL - cooked boneless chopped ham - FLAG
CC87	ST310	SRR3535391		USA - not provided
CC87	ST310	SRR5839932		USA - not provided - CDC
CC87	ST846	SRR3297278		USA - CSF - not provided
CC87	ST847	SRR1534960		USA - blood - CDC
CC87	ST87	17SEL107LM		France NRL
CC87	ST87	17SEL391LM		France NRL
CC87	ST87	CZ-NAT-SO-31		Czech Republic NRL
CC101	ST1309	ERR1940728		not provided
CC101	ST38	ERR1599782		not provided - 2014
CC101	ST101	SRR1640213		Canada - environmental - not provided
CC101	ST101	SRR6321746		Canada - frozen albacore tuna loins - 40449
CC101	ST101	SRR6966972		Canada - smoked butterfish - 36453
CC101	ST101	ERR2523710		Denmark - not provided - 2011
CC101	ST101	ERR2523746		Denmark - not provided - 2012
CC101	ST38	ERR2523790		Denmark - not provided - 2015
CC101	ST101	ERR1738821		France - Food - 1997
CC101	ST775	ERR1738673		France - Food - 2003
CC101	ST431	SRR6457860		Ireland - environmental - 2011
CC101	ST101	SRR6457848		Ireland - food - 2009
CC101	ST101	SRR6457866		Ireland - food - 2011
CC101	ST38	SRR3215348		Italy - cheese - 2011-11
CC101	ST101	SRR3823933		Italy - ricotta - 41152
CC101	ST101	SRR6321246		Japan - seasoned capelin roe - 38078
CC101	ST101	SRR6929852		Russia - herring Fish - 43173
CC101	ST101	SRR3453146		South Korea - frozen Snow Cooked Crabmeat - 42324
CC101	ST101	SRR7187041		United Kingdom:United Kingdom - human - 2016-03
CC101	ST431	SRR7163879		United Kingdom:United Kingdom - human - 2016-08
CC101	ST101	SRR1068575		USA - cheese - not provided
CC101	ST101	SRR6366165		USA:AK - environmental swabs - 39772
CC101	ST101	SRR3578980		USA:CA - environmental sponge - 38553
CC101	ST101	SRR5341551		USA:AK - environmental Swab - 42786
CC101	ST1038	SRR3065878		USA:AZ - environmental swab - 41996
CC101	ST671	GCF_003589505		UK
CC101	ST101	GCF_003588925		UK
CC101	ST101	GCF_003588185		UK
CC101	ST101	GCF_002444375		USA FDA

CC101	ST101	GCF_002028185		IE
CC101	ST101	GCF_002016665		USA FDA
CC101	ST775	SRR4301095		USA:FL - smoked salmon - 38197
CC101	ST775	ERS1375109	03EB425LM	France NRL
CC101	ST775	18SEL141LM		France NRL
CC101	ST775	18SEL144LM		France NRL
CC101	ST775	18SEL173LM	DAV161321	Luxembourg NRL
CC101	ST775	ERS1374948	AF95	France NRL
CC121	ST121	SRR6116310		North_Dakota_State_university
CC121	ST121	SRR6116326		North_Dakota_State_university
CC121	ST108	ERR1947061		not provided
CC121	ST121	SRR5120766		Germany - Cold Smoked Salmon - FDA
CC121	ST121	SRR5061760		Italy - cheese - IZS Torino
CC121	ST121	SRR3278101		Poland - bacon cut smoked salmon - FDA
CC121	ST121	SRR2015686		Poland - cold smoked salmon filet - FDA
CC121	ST236	SRR4052327		United Kingdom:South of England - human - PHE
CC121	ST121	SRR5160256		United Kingdom - retail meat - FERA
CC121	ST735	SRR2341566		USA - blood - not provided
CC121	ST716	SRR1509572		Netherlands - cervix - FDA
CC121	ST121	SRR1688449		USA:VA - smoked salmon - VA
CC121	ST1217	SRR2192200		USA - not provided
CC121	ST1043	SRR4450288		USA - not provided
CC121	ST121	11CEB362LM		France NRL
CC121	ST121	11CEB432LM		France NRL
CC121	ST121	2CEB1360LM		France NRL
CC121	ST121	12CEB861LM		France NRL
CC121	ST121	13CEB529LM		France NRL
CC121	ST121	14SEL49LM		France NRL
CC121	ST121	17SEL31LM	1484	Slovenia NRL
CC121	ST121	17SEL520LM		France NRL
CC121	ST121	17SEL521LM		France NRL
CC121	ST121	17SEL545LM		France NRL
CC121	ST121	17SEL58LM		France NRL
CC121	ST121	17SEL62LM		France NRL
CC121	ST121	17SEL63LM		France NRL
CC121	ST121	17SEL64LM		France NRL
CC121	ST121	17SEL67LM		France NRL
CC121	ST121	17SEL9LM		France NRL
CC121	ST121	16SEL1185LM		SLCC
CC121	ST121	16SEL1187LM		SLCC
CC121	ST121	16SEL1188LM		SLCC
CC121	ST121	16SEL1274LM		USA Cornell
CC121	ST121	16SEL1275LM		USA Cornell
CC121	ST121	16SEL564LM	L539	Slovenia NRL
CC121	ST121	16SEL767LM		USA Cornell
CC121	ST121	16SEL787LM	M11	North Macedonia NRL
CC121	ST121	15SEL1688LM	14/34	Ireland NRL
CC121	ST121	15SEL1648LM	14/159	Ireland NRL
CC121	ST121	17SEL25LM		France NRL
CC155	ST1307	ERR1940718		not provided
CC155	ST320	ERR1102336		not provided - human - 2013
CC155	ST155	SRR6740438		Austria - environmental/food - 2016
CC155	ST155	SRR6740454		Austria - environmental/food - 2017
CC155	ST372	SRR2075156		Canada - cooked snow crab - 40751
CC155	ST155	SRR1640183		Canada - food - 1981
CC155	ST372	SRR1378355		Canada - green sea urchin roe - 41731
CC155	ST1020	SRR5764945		Canada:Alberta - food - 33606
CC155	ST155	SRR5764975		Canada:British Columbia - food processing environment - 2009
CC155	ST155	ERR1738670		France - Food - 2003
CC155	ST155	ERR1738715		France - Food - 2007
CC155	ST155	ERR2261617		Germany - human listeriosis - 2016
CC155	ST155	ERR2261735		Germany - human listeriosis - 2017
CC155	ST155	SRR5087886		Italy - not provided - 2014
CC155	ST155	SRR3345860		Italy - salami - 2014-09
CC155	ST155	SRR5341561		Japan - seasoned squid - 37188
CC155	ST155	SRR5764977		Switzerland - sporadic listeriosis case - 2006
CC155	ST155	SRR5975721		Thailand - frozen marlin cubes - 2017-07
CC155	ST155	SRR4052256		United Kingdom:London - human - 2013-04
CC155	ST155	SRR7223194		United Kingdom:United Kingdom - human - 2018-05
CC155	ST155	SRR1566205		USA - mexican-style soft cheese - 34561
CC155	ST155	SRR1623020		USA - yellow lake perch - 33885
CC155	ST155	SRR6231891		USA:CA - environmental swabs - 41041
CC155	ST372	SRR5865510		USA:FL - bean sprouts - 37392
CC155	ST155	SRR4014393		USA:FL - beef top sirloin steak - 38628
CC155	ST155	15SEL252LM		France NRL
CC155	ST155	ERS1374944	07CEB349LM	France NRL
CC155	ST155	ERS1374994	08CEB181LM	France NRL
CC155	ST155	64AT_2018_FOOD38		Austria NRL
CC155	ST155	64NL_2016_FOOD11		Netherlands NRL
CC155	ST155	ERS1375025	03EB246LM	France NRL
CC155	ST155	14SEL1548LM		France NRL
CC155	ST155	14SEL810LM		France NRL
CC155	ST155	14SEL986LM		France NRL
CC155	ST155	17SEL412LM		France NRL
CC155	ST155	13CEB785LM		France NRL
CC193	ST796	SRR1201375		USA

CC193	ST196	ERR1100964		not provided
CC193	ST193	SRR6059701		Canada - frozen crawfish - FDA
CC193	ST662	SRR5764954		Canada:British Columbia - Food -
CC193	ST193	ERR1738826		France - Food -
CC193	ST193	SRR5085024		Italy - bovine cheese - blue cheese - IZS Torino
CC193	ST193	SRR6304499		Korea - seasoned pollack roe - FDA
CC193	ST193	SRR1509669		Viet Nam - frozen sliced trout - FDA
CC193	ST193	SRR1636540		USA:NY - smoked fish - NYAG
CC193	ST796	SRR5120762		USA:SC - environmental swabs - FDA
CC193	ST796	SRR5452683		USA:UT - environmental swab - FDA
CC193	ST796	SRR4733510		USA:MN - Hash Browns - Minnesota Department of Health
CC193	ST193	ERR1738693		France - Food -
CC193	ST662	ERS1375105	01EB168LM	France NRL
CC193	ST193	ERS1375045	06CEB178LM	France NRL
CC193	ST193	ERS1374976	06CEB180LM	France NRL
CC193	ST193	ERS1375073	08CEB03LM	France NRL
CC193	ST193	ERS1375091	11CEB245LM	France NRL
CC193	ST193	ERS1374979	IN1	France NRL
CC193	ST193	ERS1374997	IN10	France NRL
CC193	ST193	ERS1374947	IN9	France NRL
CC199	ST199	GCF_001564775		France
CC199	ST199	GCF_001751665		USA
CC199	ST199	GCF_003191505		USA
CC199	ST199	GCF_001709845		Canada
CC199	ST199	GCF_001826755		USA
CC199	ST199	GCF_003608385		USA
CC199	ST199	ERS1375041	SO56	France
CC199	ST199	GCF_003031975		USA
CC199	ST199	GCF_003668115		USA
CC199	ST199	GCF_001711475		Canada
CC199	ST199	GCF_001827225		USA
CC199	ST199	GCF_001583055		USA
CC199	ST199	GCF_002000285		China
CC199	ST199	GCF_003703675		UK
CC199	ST199	GCF_003608155		USA
CC199	ST199	GCF_001826875		USA
CC199	ST199	GCF_001759155		USA
CC199	ST199	GCF_003587695		UK
CC199	ST199	GCF_001826735		USA
CC199	ST199	16SEL689LM		France NRL
CC204	ST1299	ERR1940708		not provided
CC204	ST204	SRR5764855		Canada:British Columbia - food/environmental - 2012
CC204	ST204	SRR5883549		China - spicy seaweed salad - 42921
CC204	ST204	ERR1738770		France - food - 2011
CC204	ST204	ERR1738794		France - food - 2012
CC204	ST204	SRR3646062		Ireland - human - 2011
CC204	ST204	SRR5084468		Italy - bovine cheese - blue cheese - 2004
CC204	ST204	SRR3345952		Italy - deli meat product - 2011-11
CC204	ST204	SRR3391849		Italy - environment - 2004
CC204	ST204	SRR3579421		Japan - seasoned flying fish roe - 40074
CC204	ST204	SRR3330415		Poland - smoked salmon - 40303
CC204	ST204	SRR4052108		United Kingdom:Midlands and East of England - human - 2013-02
CC204	ST204	SRR7167739		United Kingdom:United Kingdom - human - 2016-05
CC204	ST204	SRR5297401		USA:CA - Environmental:non-food-contact surface - 2017
CC204	ST204	SRR1818029		USA:CA - grilled chicken breast - not provided
CC204	ST204	SRR2636963		USA:FL - ham sausage with veal - 41982
CC204	ST204	SRR3669853		USA:FL - pastrami salmon - 38378
CC204	ST1129	SRR5378751		USA:GA - environmental swabs - 42766
CC204	ST204	SRR1849359		USA:GA - ready-to-eat product:fully cooked chicken breast - 2014
CC204	ST204	SRR3490006		USA:RI - Food - 42283
CC204	ST204	SRR2992823		USA:WA - environmental swab - 42324
CC204	ST204	SRR3115257		USA:WA - Ice cream - 42063
CC204	ST204	ERS1374980	11CEB216LM	France NRL
CC204	ST204	ERS1375009	11CEB450LM	France NRL
CC204	ST204	ERS1374951	12CEB03LM	France NRL
CC204	ST204	18SEL140LM		France NRL
CC204	ST204	17SEL510LM		France NRL
CC204	ST204	LV0294		Czech Republic NRL
CC204	ST204	17SEL373LM		France NRL
CC224	ST845	SRR5807994		USA - blood - 2017-06
CC224	ST224	SRR5764938		Canada:British Columbia - food - 2012
CC224	ST224	ERR2523763		Denmark - not provided - 2015
CC224	ST224	ERR2523761		Denmark - not provided - 2015
CC224	ST224	SRR1509665		France - cabrioulet cheese (aged over 2 months) - 41796
CC224	ST224	ERR1738762		France - food - 2010
CC224	ST224	SRR1378348		Italy - fontina cheese - 41744
CC224	ST224	SRR3945600		Italy - gorgonzola dolce cheese - 41390
CC224	ST224	SRR3345806		Italy - salmon - 2013-05
CC224	ST224	SRR5085114		Italy - Silage Feed - 2010
CC224	ST224	SRR5764910		Switzerland - carcasses - 2011
CC224	ST224	SRR5765009		Switzerland - sporadic listeriosis case - 2005
CC224	ST224	SRR5764907		Switzerland - tuna sandwich - 2011
CC224	ST224	SRR5318941		Ukraine - fish-fillet atlantic in oil - 41178
CC224	ST224	SRR6325476		Ukraine - fish-herring fillet in oil - 40925
CC224	ST224	SRR5344710		United Kingdom - retail meat - not provided
CC224	ST224	SRR4052338		United Kingdom:London - human - 2014-10

CC224	ST386	SRR4052340		United Kingdom:North of England - human - 2013-12
CC224	ST224	SRR1535745		USA:AZ - Hass avocados - 41801
CC224	ST224	SRR3181840		USA:VA - environmental swab - 40585
CC224	ST224	SRR5526961		USA:AZ - whole avocados - 41820
CC224	ST224	SRR2143485		USA:CA - environmental swab - 42115
CC224	ST224	SRR3657444		USA:FL - whitefish salad - 38343
CC224	ST224	SRR1422640		USA:KS - drain - 41767
CC224	ST224	ERS1374999	10CEB615LM	France NRL
CC224	ST224	16SEL725LM		France NRL
CC224	ST224	18SEL186LM	DAV171058	Luxembourg NRL
CC224	ST224	17SEL397LM		France NRL
